# Prognostic models of diabetic microvascular complications: a systematic review and meta-analysis

**DOI:** 10.1186/s13643-021-01841-z

**Published:** 2021-11-01

**Authors:** Sigit Ari Saputro, Oraluck Pattanaprateep, Anuchate Pattanateepapon, Swekshya Karmacharya, Ammarin Thakkinstian

**Affiliations:** 1 Department of Clinical Epidemiology and Biostatistics, Faculty of Medicine Ramathibodi Hospital, Mahidol University, 270 Rama VI Road, Pyathai, Bangkok, 10400 Thailand; 2 Department of Epidemiology Biostatistics Population and Health Promotion, Faculty of Public Health, Airlangga University, Surabaya, Indonesia

**Keywords:** Meta-analysis, Microvascular complications, Prognostic model, Type 2 diabetes

## Abstract

**Background:**

Many prognostic models of diabetic microvascular complications have been developed, but their performances still varies. Therefore, we conducted a systematic review and meta-analysis to summarise the performances of the existing models.

**Methods:**

Prognostic models of diabetic microvascular complications were retrieved from PubMed and Scopus up to 31 December 2020. Studies were selected, if they developed or internally/externally validated models of any microvascular complication in type 2 diabetes (T2D).

**Results:**

In total, 71 studies were eligible, of which 32, 30 and 18 studies initially developed prognostic model for diabetic retinopathy (DR), chronic kidney disease (CKD) and end stage renal disease (ESRD) with the number of derived equations of 84, 96 and 51, respectively. Most models were derived-phases, some were internal and external validations. Common predictors were age, sex, HbA1c, diabetic duration, SBP and BMI. Traditional statistical models (i.e. Cox and logit regression) were mostly applied, otherwise machine learning. In cohorts, the discriminative performance in derived-logit was pooled with *C* statistics of 0.82 (0.73‑0.92) for DR and 0.78 (0.74‑0.83) for CKD. Pooled Cox regression yielded 0.75 (0.74‑0.77), 0.78 (0.74‑0.82) and 0.87 (0.84‑0.89) for DR, CKD and ESRD, respectively. External validation performances were sufficiently pooled with 0.81 (0.78‑0.83), 0.75 (0.67‑0.84) and 0.87 (0.85‑0.88) for DR, CKD and ESRD, respectively.

**Conclusions:**

Several prognostic models were developed, but less were externally validated. A few studies derived the models by using appropriate methods and were satisfactory reported. More external validations and impact analyses are required before applying these models in clinical practice.

**Systematic review registration:**

PROSPERO CRD42018105287

**Supplementary Information:**

The online version contains supplementary material available at 10.1186/s13643-021-01841-z.

## Background

Type 2 diabetes (T2D) has increased rapidly over the past 30 years becoming worldwide public health problem with prevalence in adults of 463 million (9.3%) in 2019. It is estimated to be 700 million (10.9%) by 2045 [1], in which currently about 79% of people have diabetes living in low- and middle-income countries [1, 2]. Furthermore, diabetic progression due to its complications-increased disability, impaired quality of life and leading cause of premature death, which accounted for 11.3% of the global mortality [1, 3].

Two life threatening microvascular complications in T2D are diabetic retinopathy (DR) and diabetic nephropathy (DN). DN, known as chronic kidney disease (CKD), characterised by proteinuria and rapidly declined glomerular filtration rate (GFR) [2, 4], accounted for approximately 20‑40% of diabetic populations [5]. DR is the major cause of blindness [6] through fractional retinal detachment, preretinal or vitreous haemorrhage and central vision impairment, with the prevalence of 25% globally [7].

Diabetic microvascular complications commonly occurs in working ages [8], thus declining productivity, increasing tremendous social cost and high burden in healthcare [9, 10]. Therefore, early identification of high-risk patients to prevent occurrence of microvascular complications is very important. Many prognostic models have been developed (e.g. DR [11–18], CKD [19–25] and ESRD models [12, 26–29]) using various statistical methods. A lot of prognostic models were externally validated [12, 17, 19, 20, 22, 26, 29, 30], whilst other models were not [13, 15, 16, 21, 27, 28]. Nonetheless, the best prognostic model for each complication was still inconclusive. Hence, we conducted a systematic review to summarise all prognostic models for diabetic microvascular complications (including DR, CKD and ESRD) that are available and their performances in prediction of complications.

## Methods

### Protocol registration

This study was conducted following Preferred Reporting Items for Systematic Reviews and Meta-Analyses (PRISMA) [31] and in accordance with CHARMS checklist [32]. The review protocol was registered at PROSPERO (CRD42018105287).

### Search strategy

Studies were identified from PubMed and Scopus up to 31 December 2020. Search terms were constructed based on patients, interventions and outcomes, see details in Additional files 1 and 2.

### Study selection

Studies, published in any language, were eligible if they studied in adult T2D, developed or validated any multivariable prognostic models of microvascular complications in T2D with applying any traditional statistical modelling (e.g. logit or Cox regression etcetera) or machine learning (ML), and reported model performance. We also included the studies from reference list of relevant publications.

### Data extraction

Data extractions were performed by one reviewer (SAS) and checked by OP. Extracted data were characteristics of study and patients (i.e. country, study design, settings, data source, sample size and number of events, ethnicity, age, percent male and diabetic duration), study phase (i.e. derivation or validation), statistical methods, predictors, missing data and outcomes (i.e. DR, CKD and ESRD). In addition, two related properties of model performances (calibration and discrimination) were also extracted.

### Risk of bias assessment

Risk of bias assessment was assessed by using Prediction Model Risk of Bias Assessment Tool (PROBAST) [32]. Each item was rated as low, high or unclear. The overall validity was low and high risk if all domains were low risk, and at least one domain was high risk, respectively. Discrepancies were solved by consensus between the team.

### Statistical analysis

Characteristics of each prognostic model and predictive performances (including calibration and discrimination) were described. Discrimination was assessed according to original included studies, in which C-statistic was mostly used. If studies reported C-statistic without variance, it was estimated using equations in the previous guidelines [32–34]. Calibration was assessed [35] using calibration plot, goodness-of-fit testing (i.e. Hosmer and Lemeshow *χ*^2^ test), calibration slopes or the observed/expected (O/E) ratio.

A meta-analysis was applied for pooling *C* statistics across studies stratified by study’s design/phase, statistical model and T2D complications. A random-effect model by *DerSimonian-Laird* [36, 37] was used if heterogeneity was present (*p* value < 0.10 or *I*^2^ > 25%); otherwise, a fixed-effect model was used. A heterogeneity was assessed by Cochrane *Q* test and *I*^2^ statistic. Publication bias in external validation was assessed using funnel-plot [38] and Egger’s test [39]. All statistical analyses were performed using STATA 16 [40]. A *p* value less than 0.05 was considered as statistically significant, except for heterogeneity which used 0.10.

## Results

A total of 32/1009 and 44/3321 studies were eligible for DR and DN, respectively, see Table S1 (Figs. S1‑S2). Amongst them, 205 prognostic equations were derivative, some of them performed internal and external validations. Most studies reported *C* statistics, but only a few-portions reported calibrations (Table S2).

### Risk of bias assessment

Risk of bias assessment of all included studies was presented in Table S3. Amongst 71 studies, about 86 to 95% of studies were determined as low risk of bias for study participants, selection of predictors and outcome measurement. About 23% and 40% of studies were rated as high risk for sample size, participant flow and statistical analysis, respectively. As a result, 35% of studies were overall low risk of bias (Fig. S3).

### Diabetic retinopathy

Thirty-two [11–18, 24, 30, 41–62] studies were identified for predictions of DR including 1,120,278 diabetic patients with 128,129 (11.4%) events. Of which, 26 [11, 12, 14, 18, 24, 30, 41 50, 53, 62] and 6 [13, 15–17, 51, 52] studies applied traditional statistical modelling and ML, respectively. Twenty-eight [11–18, 24, 41, 42, 45, 47–62] studies derived 84 original prognostic equations with varied sample sizes of 18 to 254,896. Mean age was 44.6 to 66.6 years, percent male was 27.0 to 61.9, and diabetic duration varied from 1.4 to 15.8 years. Most studies were conducted in Europe and America regions with only 11 [17, 42, 43, 45, 47, 49, 52–55, 59] (34%) studies in Asia. Twenty-four [11–16, 18, 24, 30, 41–43, 45–48, 51, 52, 54, 56, 58–60, 62] and 8 [17, 44, 49, 50, 53, 55, 57, 61] studies were hospital and community-based settings respectively with confirmation of T2D diagnosis from medical records, laboratory tests or use of diabetic drugs. DR was mostly diagnosed by using fundus examination. Follow-up time ranged varied from 1.0 [56] to 20 [11] years with a median of 5 years. Only 5 [18, 24, 30, 50, 58] studies reported percent loss to follow-up which ranged from 2.4 to 31.3%. Eighteen [11–13, 15, 30, 41–45, 48, 50, 51, 54, 55, 57, 59, 61] studies used various methods for dealing with missing data, in which about a half of them used multiple imputations (Table S1). Four [45, 47, 54, 57] and 3 [13, 18, 49] studies provided simplified scoring system and presented nomograms, or else only used regression coefficients/odds ratio to calculate the score.

As for phase of prediction, 4 [12, 17, 41, 42], 2 [11, 45], 4 [30, 43, 44, 46], 15 [13, 15, 16, 18, 48-54, 56‑59] and 6 [14, 24, 55, 60, 62, 63] studies were respectively determined as derived-internal-external (D/I/E), derived-external (D/E), external (E), derived-internal (D/I) and only derived (D) phases (Table S2). Amongst 8 [12, 17, 30, 41–44, 46] external-validation studies, 5 [12, 17, 41, 42, 44] validated their own derived models in the same ethnicity (i.e. Asian [17, 42], mixed ethnicity [12, 41, 44]), except 3 [30, 43, 46] that validated other author’s models that were originally in Caucasians [11] and Asians [45, 47]. Most studies were cohorts/RCT’s, and their DR incidence varied from 1.5% [48] to 42.9% [56] whereas it was 14.2% [43] to 57.2% [54] in cross-sectional studies.

Seventeen [13, 14, 17, 42, 43, 45, 47, 49, 51–56, 58, 61, 64] and 9 [11, 12, 18, 24, 41, 44, 46, 59, 60] studies used logit and Cox whereas 6 [13, 15–17, 51, 52] applied MLs. Various predictors were considered (Fig. S4), in which the most commonly used were diabetic duration, age, HbA1c, SBP and BMI, which these were mainly included in the models as continuous predictors. A total number of included predictors in the conventional statistical models and MLs were not much different with a median of 8 (range 2‑37). Interestingly, few studies used image/signal analytic [56, 58] and genetic variables [42, 55, 62], which were incorporated with conventional clinical data. Two [56, 58] studies predicted specific DR site using multifocal electroretinogram incorporated with traditional clinical factors [56, 58].

Three [42, 55, 62] studies derived genetic risk score (GRS) based on different genetic polymorphisms (range 2‑76). Traditional prognostic factors (i.e. age, sex, diabetic duration, HbA1c and hypertension/SBP) were also retained in the model with GRS.

*C* statistic varied from 0.50 [13] to 0.95 [58], 0.52 [17] to 0.92 [58] and 0.59 [12] to 0.83 [30] for derived, internal and external validations. Those prognostic equations had been externally validated with moderate to good performance (Table S4). Discrimination performance of logit equations varied from moderate to high with the *C* statistics of 0.70 [45, 47, 49, 62] to 0.95 [58] and 0.63 [47] to 0.92 [58] in derived and internal validation; likewise, for support vector ML [17] in these corresponding phases of 0.83 and 0.81.

**Fig. 1:**
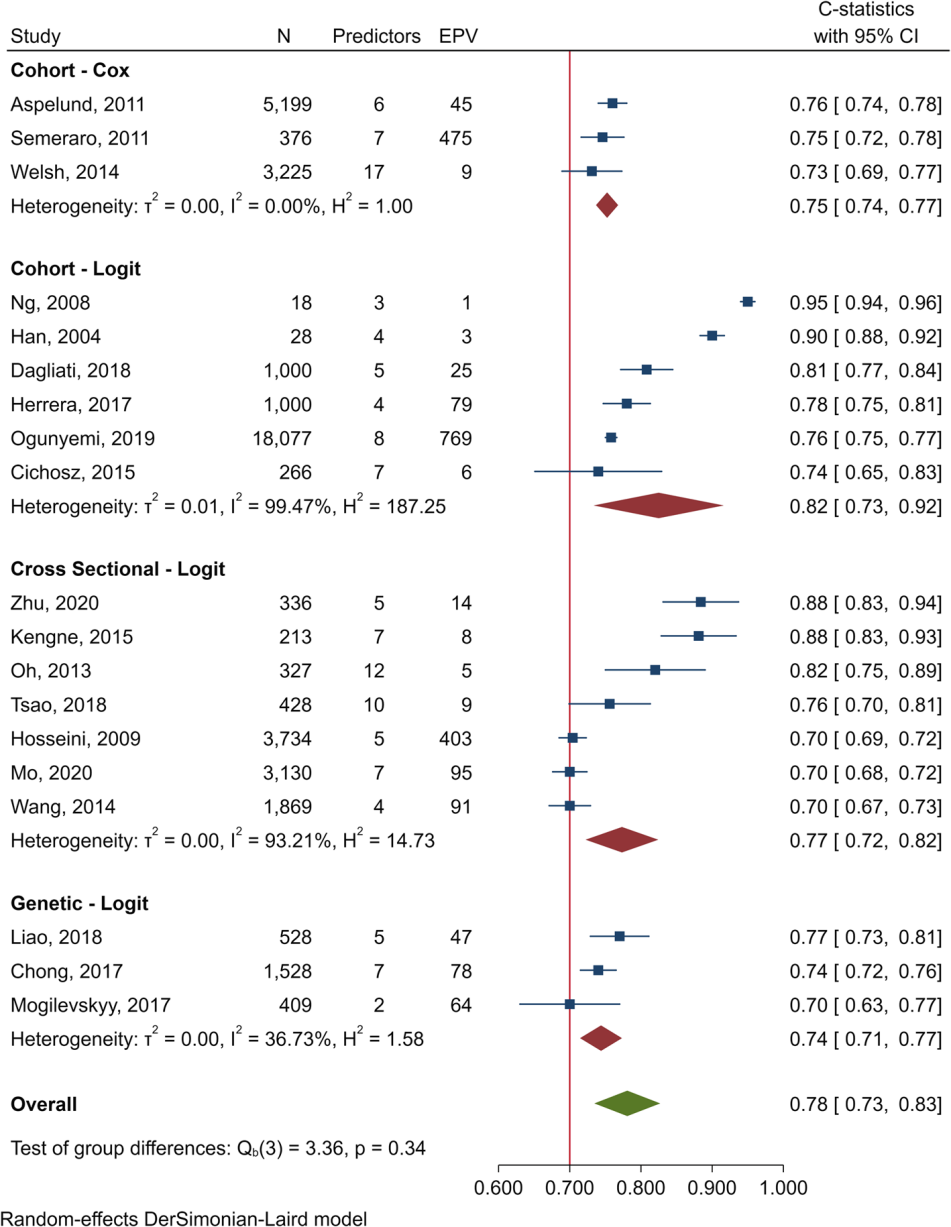
Forest plot of *C* statistics in derivative phase for DR

Pooled *C* statistics of the derived-logit models across cohorts [13, 51, 56–58, 61], cross-sectional [14, 17, 45, 47, 49, 52, 53] and case-control genetic [42, 55, 62] studies were 0.82 (0.73‑0.92; *I*^2^ = 99.47%), 0.77 (0.72‑0.82; *I*^2^ = 93.21%) and 0.74 (0.71‑0.77; *I*^2^ = 36.73%), respectively (Fig. 1). Fixed effect model was observed on pooled derived-Cox [11, 18, 24] models in cohort studies which yielded 0.75 (0.74‑0.77; *I*^2^ = 0.0%).

Pooled *C* statistics of logit equations across cohorts [13, 51, 56–58] and cross-sectional [17, 47, 49, 52–54] studies for internal validation were 0.83 (0.76‑0.90; *I*^2^ = 95.11%) and 0.74 (0.68‑0.81; *I*^2^ = 82.46%), respectively. Of those, the external validation for three [17, 45, 47] equations yielded 0.81 (0.78‑0.83; *I*^2^ = 8.05%) by cross-sectional studies of logit regression (Table S5). Funnel plot and Egger’s test (*p* > 0.293) suggested no publication bias by the absence of small study effects, no correlation between sample size and the magnitude of *C* statistics in external validation studies (Fig. S5).

Model’s calibration in derived phase [11, 12, 14, 18, 49, 54, 59, 61], internal [12, 18, 41, 49, 54, 59] and external [12, 30, 41, 44, 46] validations mostly demonstrated perfect O/E. Nine studies [14–17, 24, 52, 56, 58, 61] might have overfitted model as the ratio of an event per variable (EPV) numbers ranged from 1 [58] to 9 [24, 52], whereas the other 2 [12, 45] models might have underfitted with a ratio as high as 297 [12] to 403 [45].

### Diabetic nephropathy

#### CKD

Thirty [12, 13, 19–25, 44, 59, 65–83] studies purely derived 96 equations including 244,934 diabetic patients with 44,023 (17.9%) events of CKD. CKD incidence ranged from 12.1 to 37.3% for 5 RCT’s [12, 59, 70, 74, 77], and 0.7% to 47.6% for 22 [12, 13, 20, 22–25, 65, 66, 68, 69, 71–73, 75, 76, 78–83] cohorts. Eleven studies [19, 22, 25, 59, 65–67, 69, 76, 78, 82] (36.7%) were conducted in Asians. Sixteen [13, 21, 23, 66–69, 71, 72, 75, 76, 78, 79, 81–83] and 16 [12, 19, 20, 22, 24, 25, 44, 59, 65, 69–71, 73, 74, 77, 80] studies diagnosed CKD based on eGFR and albuminuria, respectively. Median (range) follow-up time was 5.4 (1‑10) years and percent lost to follow up was 0.8% to 35.7%. Twelve [12, 13, 20, 22, 25, 44, 59, 65, 66, 69, 73, 80] (38.7%) studies reported methods for dealing with missing data, 5 [12, 22, 44, 59, 80] had used multiple imputations and only a few reported percent missing data [12, 13, 20, 44].

Five [12, 19, 23, 68, 71], 2 [20, 25], 2 [44, 69], 12 [13, 22, 59, 65–67, 70, 72–74, 78, 79] and 9 [21, 24, 75–77, 80–83] studies were D/I/E, D/E, E, D/I and only D-phases, respectively. Of 9 E-phases, eight [12, 19, 20, 23, 25, 44, 68, 71] studies validated their own models in different datasets, and one [69] validated others author’s model (i.e. QKIDNEY risk score), which developed in general populations. Half of the studies were validated in Asians [19, 20, 25, 43], and 9 [12, 20, 23, 25, 44, 68, 69, 71] used data from cohorts/RCTs. Their mean age ranged from 44.0 to 67.3 years, whereas the percent of male varied from 32.5 to 76.0 with a median follow-up time of 4.9 years.

Out of 28 studies, 96 derived models consisted of 79 traditional statistical models (i.e. logit (*n* = 16) [13, 19-21, 23, 25, 65‑68, 70, 72, 73, 78, 79, 82] and Cox (*n* = 11) [12, 22, 24, 59, 74–77, 80, 81, 83]), whereas 17 models (*n* = 4) [13, 70, 71, 73] performed various MLs algorithms (Table S2). Three [13, 65, 66] studies provided nomograms, whereas 2 [25, 74] studies simplified risk score.

Ninety-two derived models reported *C* statistics, with 55 (59.7%) internal and 19 (20.6%) external validations. Their discriminative performance varied from 0.50 [13] to 0.93 [21, 66], 0.50 [13] to 0.91 [73] and 0.57 [19] to 0.85 [44] in derived, internal and external phases, respectively (Table S2), which were explicitly described (Table S4). Common predictors were SBP, HbA1c, sex, diabetic duration and eGFR. Two [19, 67] studies combined genetic factors with clinical factors which yielded better discrimination of 0.78 (0.75‑0.81) relative to considered conventional models 0.75 (0.72‑0.78), see Table S6.

Out of twenty-eight [12, 13, 19–25, 59, 65–68, 70–83] derived studies, 14 [20, 22, 23, 59, 65–68, 72–74, 78–80] studies reported acceptable calibration model with O/E ratio ranged from 0.77 [79] to 1.11 [73]. Only seven [12, 23, 67, 73, 74, 78, 79] and six [12, 20, 22, 44, 68, 69] out of 21 validated-studies had O/E ratio of 0.93 [78] to 1.14 [12] and 0.97 [44] to 1.31 [12] in I and E phases (Table S2).

**Fig. 2:**
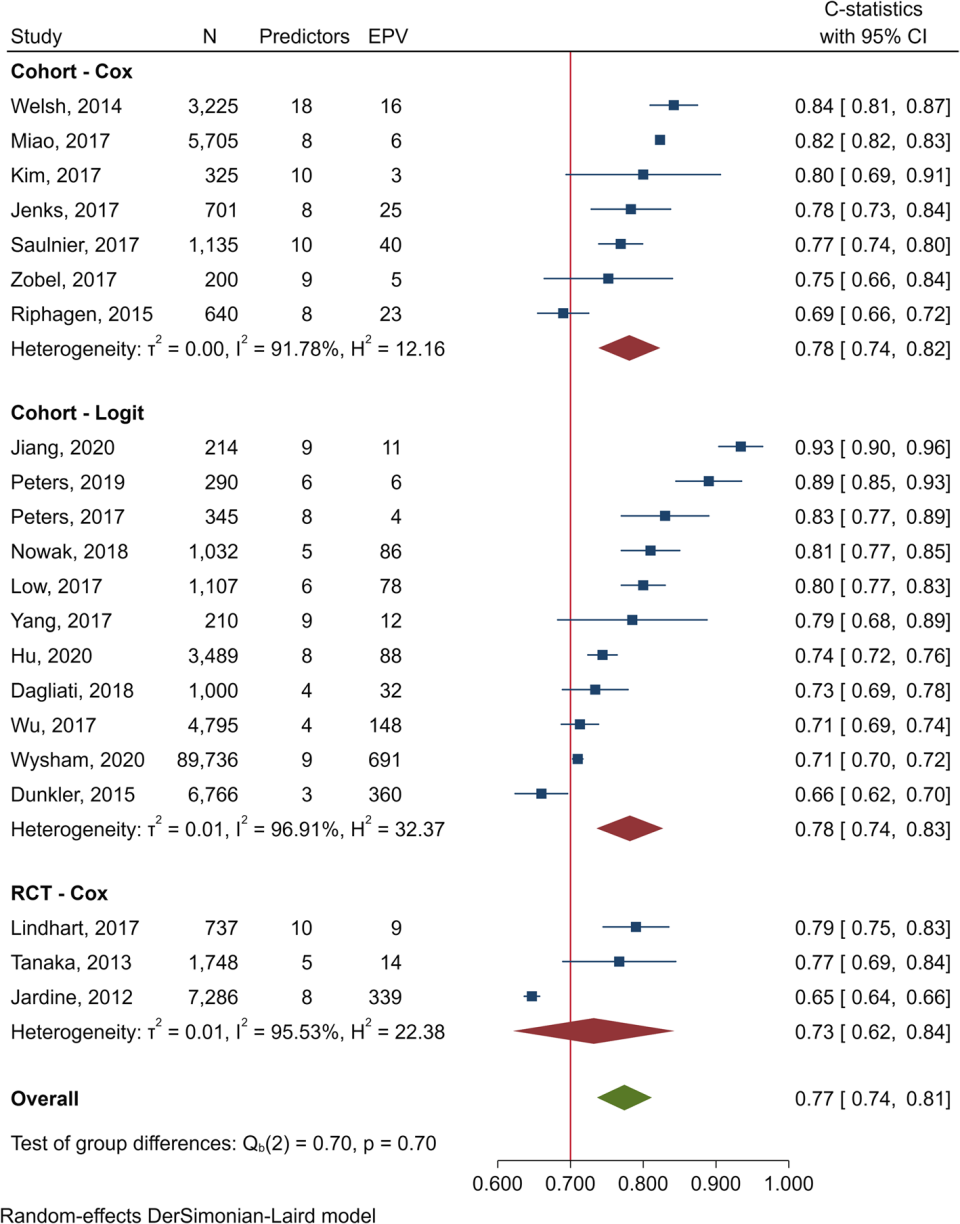
Forest plot of *C* statistics in derivative phase for CKD

For cohorts, the pooled *C* statistics for logit (*n* = 11) [13, 20, 23, 25, 65, 66, 68, 72, 78, 79, 82] and Cox (*n* = 7) [22, 24, 75, 76, 80, 81, 83] in D phase were 0.78 (0.74‑0.83; *I*^2^ = 96.91%) and 0.78 (0.74‑0.82; *I*^2^ = 91.78%), respectively (Fig. 2). Cox regression in derived RCTs (*n* = 3) [59, 74, 77] yielded pooled *C* statistics of 0.73 (0.62‑0.84; *I*^2^ = 95.53%).

Derived logit (*n* = 8) [13, 23, 65, 66, 68, 72, 78, 79] from cohorts were internally validated yielding the pooled *C* statistics of 0.79 (0.74‑0.83; *I*^2^ = 95.53%) which performed closely to the D phases, but poorer in externally validated [20, 23, 25, 68] with pooled *C* statistic of 0.75 (0.67‑0.84; *I*^2^ = 94.68%). Funnel plot and *Egger’s* test (*p* > 0.710) showed no publication bias by the absence of small study effects (Fig. S6). There is no correlation between studies for smaller cohorts with higher *C* statistics.

#### ESRD

Eighteen studies [12, 26–29, 44, 72, 74, 84–93] originally derived 46 models in 366,210 diabetic patients with the ESRD incidence of 57,294 (15.65%). Of them, 13 [26, 28, 29, 44, 72, 84–87, 89–92] and 5 [12, 27, 74, 88, 93] were cohorts and RCTs, respectively. A half of them were conducted in Asia [28, 29, 84, 85, 89, 90, 92] and the USA [12, 27, 44, 72, 86, 87, 91]. Thirteen [12, 26–29, 72, 74, 84, 88–91, 93] and 5 [44, 85–87, 92] studies were hospital-based and community-based settings respectively, where ESRD was mostly confirmed by dialysis [12, 26–28, 44, 72, 88–92]. Mean follow-up times ranged from 1.5 to 14 years. Only 9 studies (50%) reported methods for dealing with missing data, in which 5 [12, 27, 29, 44, 84] used multiple imputations. Two studies [74, 84] developed simplified risk score based on the Framingham Heart study [94].

Briefly, 1 [12], 2 [26, 29], 1 [44], 5 [28, 72, 74, 84, 85] and 9 [27, 86–93] studies showed D/I/E, D/E, E, D/I, and only D phases, respectively (Table S1). Three [12, 26, 44] studies have externally validated their own models within the same studies, whereas 1 [29] study validated other models’ studies [26, 74].

*C* statistics varied from 0.76 [89] to 0.97 [27, 85] in derivative phases of 17 studies (*n* = 51), 0.60 [12] to 0.96 [85] in internal validations (*n* = 11) and 0.54 [12] to 0.92 [26] in external validations (*n* = 13), see Table S2. Prognostic model for ESRD was mainly derived by Cox equation in 16 (95%) studies [12, 26‑29, 74, 84-93].

**Fig. 3:**
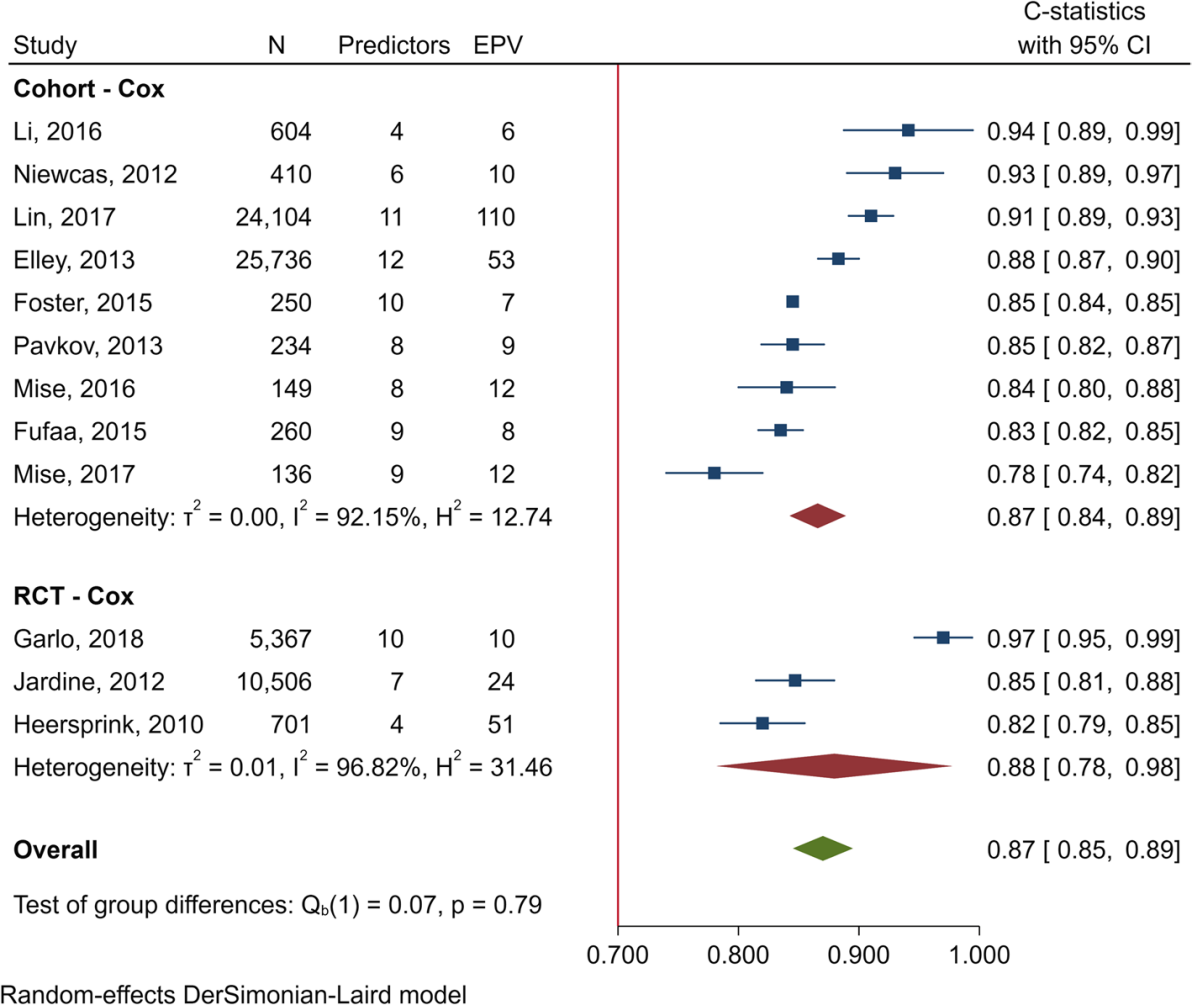
Forest plot of *C* statistics in derivative phase for ESRD

The pooled *C* statistics in cohorts using Cox were 0.87 (0.84‑0.89; *I*^2^ = 92.15%), 0.91 (0.86‑0.96; *I*^2^ = 94.86%) for derived and internal validations suggesting discrimination in validations were not much different compared with derived phases. The pooled *C* statistics in derived RCTs were 0.88 (0.78‑0.98; *I*^2^ = 96.82%), see Fig. 3. Moreover, pooled *C* statistics in external validation demonstrated good performance of 0.86 (0.85‑0.88) in 3 [26, 29, 44] cohorts (Table S5). Funnel plot and Egger’s test (*p* > 0.513) showed no publication bias by the absence of small study effects in external validation studies for predicting ESRD (Fig. S7).

Common predictors for ESRD were age, sex, HbA1c, eGFR and BMI (Table S6). Predictive models of 6 [27, 28, 86, 87, 91] studies might have over-fitted as events/variable for applying Cox resulted in 6 [28] to 9 [92], whereas their ratios in 4 [12, 29, 89, 90] other studies observed a rule of thumb of 10‑20 (Fig. 3, Table S2).

## Discussion

This review summarised prognostic models that were developed and validated for predicting microvascular complications (i.e. DR, CKD and ESRD) in T2D patients. Model performances were described prognostic models separately by derived, internal and external validation.

Seven predictors were commonly used in predictive models of DR, DN and ESRD including age, sex, BMI, diabetic duration, HbA1c, SBP and eGFR. The DR models showed well discriminated with pooled *C* statistics of 0.82, 0.83 and 0.81 in D, I and E validations, respectively. Model performance was only moderate‑good in CKD for all phases (i.e. the corresponding pooled *C* statistics about 0.78, 0.79 and 0.75) but quite excellence for ESRD models (i.e. 0.87, 0.91 and 0.86, respectively).

Only a few prognostic models were externally validated with moderate to good discrimination performance, which are applicable in clinical practice. For instance, a few DR-models [11, 12, 41] had good discrimination and calibration in external validations. Three [12, 20, 68] DN models had good discrimination with fair calibration. Other three [12, 26, 29] ESRD models with very large size cohorts were generalisable with good discriminations and were even developed in different ethnicities. Calibration performance was less reported relative to discrimination, although both parameters should be reported for prognostic model development [95–97]. Particularly for observed to expected (O/E) ratio was reported in very few studies, which prevented meta-analysis of calibration.

Currently many prediction models are available by online calculators, or differently presented simplified risk scores or nomograms. Some online risk-calculators have been developed to simplify knowledge translation in clinical practice (i.e. DR [11, 12, 41, 59], CKD [12, 20] and ESRD [26]). However, very few of them have been applied due to the absence of some predictors and users’ interpretations in routine health practice.

We found various clinical settings and developed equations, but only few of them were externally validated with insufficiently reported with a wide range of CKD definitions. Amongst them, there might be potentially over-optimistic as EPV was less than ten by the rule of thumb in a regression model. None of the studies performed impact assessments by applying prognostic models into clinical practice.

Numerous predictors were simultaneously included in the prognostic models with a median of 8 (IQR, 5‑10) predictors. In brief, demographics, biomarkers and clinical features were commonly considered for derived-models of micro-vascular complications. Medical treatment (including anti-hypertensive and diabetic drug control) and some comorbidities were included into some derived equations. Likewise, DR itself might be a predictor of DN [20, 22, 66, 74, 75]. Interestingly, nonconventional predictors (i.e. genetic and image processing [56, 58]) could also predict DR [42, 55, 62] and DN [19, 67].

Missing data in clinical settings particularly for routine datasets are unavoidable. Frequently, the investigators only performed complete-case analysis. Handling missing data is vitally important to prevent biassed results and lost power in generalisations [98]. Additionally, categorisation of continuous predictors or dichotomisation may result in missing information, significant misleading [99], incorrect variable selection and may decrease prediction accuracy [100, 101].

Cohort or RCT should be the most appropriated design for developing prognostic model, whereas a cross-sectional study could be used for external validation. Exceptionally, nested case-control and case-cohort studies were still applicable [96]. The rule of thumb suggested that a number of 10‑20 events should be available for one predictor in a multivariable logit/Cox regression [96, 102, 103]. For instance, seven [14, 17, 24, 52, 56, 58, 61] studies in DR had EPV ratio of 1 [58] to 9 [24, 52], which might cause overfitted model. In DN, eight [21, 22, 68, 73, 76, 77, 79, 83] and seven [27, 28, 85–87, 91, 92] studies might be over-optimistic with the EPV ratio less than 10 for CKD and ESRD, respectively. Overfitting may result in poorer performance in external validation compared with derived-performance. As a result, performances of the traditional statistical models (i.e. logit, Cox) were quite varied across studies. However, ML may be better particularly when predictors themselves have collinearity and high-dimensional interaction amongst predictors. With the rapid era of big data, digitalisation and modern electronic medical records may increase used of ML techniques in derived and validation model.

As the backbone of big data analysis, ML provides the new insight and valuable algorithm in which traditional statistical models are often inadequate. Likewise, using image/signal [56, 58] analysis for predicting DR, some investigators also applied classical ML (e.g. decision trees, random forest, Naïve Bayes and neural network) to predict DN [13, 70, 73]. Nonetheless, the results of ML are black boxes, which are often difficult to interpret due to its characteristics and algorithm complexities [104, 105].

Few other factors may also influence on external-validation performance, e.g. availability of predictors, sources of data (i.e. primary data collection, survey-data or administrative/hospital-claims data), outcome rate and assessment and also population characteristics. However, only about 20 studies (25% of derived models) were externally validated. We therefore strongly suggest that those derived models should be externally validated or updated models where appropriate. Then, impact analysis should next be performed to be more confident in applying in clinical practice.

## Conclusions

This study was conducted to systematically review prognostic models of diabetic microvascular complications. Weaknesses and strengths of those prognostic models for each complication were described and commented. Some prognostic models for microvascular complications were good in discrimination in external validations, but in practice none of them performed clinical impact. The existing prognostic models for DR and CKD still need further external validation or update where appropriate. In addition, the new prognostic models should be derived using ML techniques to improve prognostic performance where required.

## Supplementary Information

**Additional file 1:** Search strategy & results from PubMed database.

**Additional file 2:** Search strategy & results from Scopus database.

**Additional file 3: Table S1.** Characteristics of studies included in systematic review. **Table S2.** Describe discrimination and calibration performances of prognostic models. **Table S3.** Risk of bias assessment using PROBAST. **Table S4.** Prognostic equations that were externally validated. **Table S5.** Summary of pooled C-statistic of prognostic model. **Table S6.** Describe variables that were included in the derivative equations.

**Additional file 4: Figure S1.** PRISMA diagram for diabetic retinopathy. **Figure S2.** PRISMA diagram for diabetic nephropathy. **Figure S3.** Risk-of-bias using PROBAST. **Figure S4.** Overview top 20 predictors included in derived models. **Figure S5.** Funnel plot (C-statistics) for DR in external validation. **Figure S6.** Funnel plot (C-statistics) for CKD in external validation. **Figure S7.** Funnel plot (C-statistics) for ESRD in external validation.

## Data Availability

All data included in this study were available in the supplementary information files.

## Abbreviations

BMI: Body mass index
CHARMS: Checklist for critical Appraisal and data extraction for systematic Reviews of prediction Modelling Studies
CKD: Chronic kidney disease
D: Derivative phase
DN: Diabetic nephropathy
DR: Diabetic retinopathy
E: External validation phase
eGFR: Estimated glomerular filtration rate
EPV: Event per variables
ESRD: End-stage renal disease
GRS: Genetic risk score
HbA1c: Glycated haemoglobin
I: Internal validation phase
ML: Machine learning
PRISMA: Preferred Reporting Items for Systematic Reviews and Meta-Analysis PROBAST Prediction model risk of bias assessment tool
RCT: Randomised controlled trials
SBP: Systolic blood pressure
T2D: Type 2 diabetes
