# Supplementary material for: Prognostic models of diabetic microvascular complications: a systematic review and meta-analysis
Source: Syst Rev. 2021 Nov 1;10:288. doi: 10.1186/s13643-021-01841-z (PMC8561867; doi:10.1186/s13643-021-01841-z)
Supplement: Supplementary file 1 — Additional file 1: Search strategy & results from PubMed database. [file 13643_2021_1841_MOESM1_ESM.docx]

## **Additional file 1. Search strategy & results from PubMed database**

| Domains | Search Code | Terms | Results |
| --- | --- | --- | --- |
| Populations (P) | #1 | Search diabetes | 792,375 |
|  | #2 | Search "diabetes mellitus" | 512,848 |
|  | #3 | Search diabetic | 792,375 |
|  | #4 | Search (((diabetic) OR "diabetes mellitus")) OR diabetes  **#1 OR #2 OR #3** | 792,375 |
| Intervention and Comparator  (IC) | #5 | Search "risk score" | 1,445,377 |
|  | #6 | Search "risk model" | 590,024 |
|  | #7 | Search "risk prediction" | 352,507 |
|  | #8 | Search "risk prognostic" | 101,194 |
|  | #9 | Search (((("risk score") OR "risk prognostic") OR "risk assessment") OR "risk model") OR "risk prediction" | 1,804,039 |
|  | #10 | Search predicting | 1,515,915 |
|  | #11 | Search "prediction tool" | 74,472 |
|  | #12 | Search "prediction rule" | 13,195 |
|  | #13 | Search "predictive model" | 535,813 |
|  | #14 | Search "prediction model" | 535,813 |
|  | #15 | Search ((((predicting) OR "prediction tool") OR "prediction rule") OR "predictive model") OR "prediction model" | 1,515,915 |
|  | #16 | Search "clinical decision model" | 42,717 |
|  | #17 | Search "clinical decision rule" | 13,195 |
|  | #18 | Search ("clinical decision model") OR "clinical decision rule" | 46,659 |
|  | #19 | Search (((((("risk score") OR "risk model") OR "risk prediction") OR "risk prognostic")) OR (((((predicting) OR "prediction tool") OR "prediction rule") OR "predictive model") OR "prediction model")) OR (("clinical decision rule") OR "clinical decision model")  **#9 OR #15 OR #18** | 2,990,940 |
| O1 | #26 | Search nephropathy | 599,945 |
|  | #27 | Search microalbuminuria | 9,203 |
|  | #28 | Search macroalbuminuria | 1,807 |
|  | #29 | chronic kidney diseases[MeSH Terms] | 117,774 |
|  | #30 | Search renal failure[MeSH Terms] | 177,065 |
|  | #31 | Search end stage kidney diseases[MeSH Terms] | 94,737 |
|  | #32 | Search (((((nephropathy) OR chronic kidney diseases [MeSH Terms]) OR microalbuminuria) OR macroalbuminuria) OR renal failure [MeSH Terms]) OR end stage renal diseases [MeSH Terms]  **#26 OR #27 OR #28 OR #29 OR #30 OR #31** | 604,505 |
| O2 | #33 | Search retinopathy  **#33** | 164,495 |
| Populations (P), Interventions –Comparator (IC), and Outcome (O) | #34 | Search (((((diabetes) OR diabetic) OR "diabetes mellitus")) AND ((((((("risk score") OR "risk model") OR "risk prediction") OR "risk prognostic")) OR (((((predicting) OR "prediction tool") OR "prediction rule") OR "predictive model") OR "prediction model")) OR (("clinical decision rule") OR "clinical decision model"))) AND retinopathy | 763 |
|  | #35 | Search (((((diabetes) OR diabetic) OR "diabetes mellitus")) AND ((((((("risk score") OR "risk model") OR "risk prediction") OR "risk prognostic")) OR (((((predicting) OR "prediction tool") OR "prediction rule") OR "predictive model") OR "prediction model")) OR (("clinical decision rule") OR "clinical decision model"))) AND ((((((nephropathy) OR chronic kidney diseases[MeSH Terms]) OR microalbuminuria) OR macroalbuminuria) OR renal failure[MeSH Terms]) OR end stage renal diseases[MeSH Terms]) | 2,348 |
