# Supplementary material for: Prognostic models of diabetic microvascular complications: a systematic review and meta-analysis
Source: Syst Rev. 2021 Nov 1;10:288. doi: 10.1186/s13643-021-01841-z (PMC8561867; doi:10.1186/s13643-021-01841-z)
Supplement: Supplementary file 2 — Additional file 2: Search strategy & results from Scopus database. [file 13643_2021_1841_MOESM2_ESM.docx]

## **Additional file 2. Search strategy & results from Scopus database**

| Domains | Search Code | Terms | Results |
| --- | --- | --- | --- |
| Populations (P) | #1 | TITLE-ABS-KEY ( diabetes ) | 925,068 |
|  | #2 | TITLE-ABS-KEY ( "diabetes mellitus" ) | 765,290 |
|  | #3 | TITLE-ABS-KEY ( diabetic ) | 394,012 |
|  | #4 | (TITLE-ABS-KEY ( diabetes ) )  OR  ( TITLE-ABS-KEY ( "diabetes mellitus" ) )  OR  ( TITLE-ABS-KEY ( diabetic ) )  **#1 OR #2 OR #3** | 1,019,419 |
| Intervention and Comparator  (IC) | #5 | TITLE-ABS-KEY ( "risk score" ) | 27,804 |
|  | #6 | TITLE-ABS-KEY ( "risk prediction" ) | 14,006 |
|  | #7 | TITLE-ABS-KEY ( "risk prognostic" ) | 380 |
|  | #8 | TITLE-ABS-KEY ( "risk model" ) | 14,962 |
|  | #9 | ( TITLE-ABS-KEY ( "risk score" ) )  OR  ( TITLE-ABS-KEY ( "risk prediction" ) )  OR  ( TITLE-ABS-KEY ( "risk prognostic" ) )  OR  ( TITLE-ABS-KEY ( "risk model" ) ) | 53,463 |
|  | #10 | TITLE-ABS-KEY ( predicting ) | 511,323 |
|  | #11 | TITLE-ABS-KEY ( "prediction tool" ) | 7,236 |
|  | #12 | TITLE-ABS-KEY ( "prediction rule" ) | 2,951 |
|  | #13 | TITLE-ABS-KEY ( "predictive model" ) | 51,117 |
|  | #14 | TITLE-ABS-KEY ( "prediction model" ) | 83,630 |
|  | #15 | ( TITLE-ABS-KEY ( predicting ) )  OR  ( TITLE-ABS-KEY ( "prediction tool" ) )  OR  ( TITLE-ABS-KEY ( "prediction rule" ) )  OR  ( TITLE-ABS-KEY ( "predictive model" ) )  OR  ( TITLE-ABS-KEY ( "prediction model" ) ) | 628,266 |
|  | #16 | TITLE-ABS-KEY ( "clinical decision model" ) | 69 |
|  | #17 | TITLE-ABS-KEY ( "clinical decision rule" ) | 1,458 |
|  | #18 | ( TITLE-ABS-KEY ( "clinical decision model" ) )  OR  ( TITLE-ABS-KEY ( "clinical decision rule" ) ) | 1,523 |
|  | #19 | ( ( TITLE-ABS-KEY ( "risk score" ) )  OR  ( TITLE-ABS-KEY ( "risk prediction" ) )  OR  ( TITLE-ABS-KEY ( "risk prognostic" ) )  OR  ( TITLE-ABS-KEY ( "risk model" ) ) )  OR  ( ( TITLE-ABS-KEY ( predicting ) )  OR  ( TITLE-ABS-KEY ( "prediction tool" ) )  OR  ( TITLE-ABS-KEY ( "prediction rule" ) )  OR  ( TITLE-ABS-KEY ( "predictive model" ) )  OR  ( TITLE-ABS-KEY ( "prediction model" ) ) )  OR  ( ( TITLE-ABS-KEY ( "clinical decision model" ) )  OR  ( TITLE-ABS-KEY ( "clinical decision rule" ) ) )  **#9 OR #15 OR #18** | 671,696 |
| O1 | #20 | TITLE-ABS-KEY ( nephropathy ) | 90,733 |
|  | #21 | TITLE-ABS-KEY ( microalbuminuria ) | 17,315 |
|  | #22 | TITLE-ABS-KEY ( macroalbuminuria ) | 2,681 |
|  | #23 | TITLE-ABS-KEY ( "chronic kidney disease" ) | 73,234 |
|  | #24 | TITLE-ABS-KEY ( "end stage kidney disease" ) | 4,168 |
|  | #25 | TITLE-ABS-KEY ( "renal failure" ) | 113,396 |
|  | #26 | ( TITLE-ABS-KEY ( nephropathy ) )  OR  ( TITLE-ABS-KEY ( microalbuminuria ) )  OR  ( TITLE-ABS-KEY ( macroalbuminuria ) )  OR  ( TITLE-ABS-KEY ( "chronic kidney disease" ) )  OR  ( TITLE-ABS-KEY ( "end stage kidney disease" ) )  OR  ( TITLE-ABS-KEY ( "renal failure" ) )  **#26 OR #27 OR #28 OR #29 OR #30 OR #31** | 267,626 |
| O2 | #27 | TITLE-ABS-KEY ( retinopathy )  **#33** | 85,988 |
| Populations (P), Interventions –Comparator (IC), and Outcome (O) | #28 | ( ( TITLE-ABS-KEY ( diabetes ) )  OR  ( TITLE-ABS-KEY ( "diabetes mellitus" ) )  OR  ( TITLE-ABS-KEY ( diabetic ) ) )  AND  ( ( ( TITLE-ABS-KEY ( "risk score" ) )  OR  ( TITLE-ABS-KEY ( "risk prediction" ) )  OR  ( TITLE-ABS-KEY ( "risk prognostic" ) )  OR  ( TITLE-ABS-KEY ( "risk model" ) ) )  OR  ( ( TITLE-ABS-KEY ( predicting ) )  OR  ( TITLE-ABS-KEY ( "prediction tool" ) )  OR  ( TITLE-ABS-KEY ( "prediction rule" ) )  OR  ( TITLE-ABS-KEY ( "predictive model" ) )  OR  ( TITLE-ABS-KEY ( "prediction model" ) ) )  OR  ( ( TITLE-ABS-KEY ( "clinical decision model" ) )  OR  ( TITLE-ABS-KEY ( "clinical decision rule" ) ) ) )  AND  ( ( TITLE-ABS-KEY ( nephropathy ) )  OR  ( TITLE-ABS-KEY ( microalbuminuria ) )  OR  ( TITLE-ABS-KEY ( macroalbuminuria ) )  OR  ( TITLE-ABS-KEY ( "chronic kidney disease" ) )  OR  ( TITLE-ABS-KEY ( "end stage kidney disease" ) )  OR  ( TITLE-ABS-KEY ( "renal failure" ) ) ) | 1,863 |
|  | #29 | ( ( TITLE-ABS-KEY ( *diabetic ) )  OR  ( TITLE-ABS-KEY ( *diabetes ) )  OR  ( TITLE-ABS-KEY ( "diabetes mellitus" ) ) )  AND  ( ( ( TITLE-ABS-KEY ( "risk score" ) )  OR  ( TITLE-ABS-KEY ( "risk model" ) )  OR  ( TITLE-ABS-KEY ( "risk prediction" ) )  OR  ( TITLE-ABS-KEY ( "risk prognostic" ) ) )  OR  ( ( TITLE-ABS-KEY ( predicting ) )  OR  ( TITLE-ABS-KEY ( "prediction tool" ) )  OR  ( TITLE-ABS-KEY ( "prediction model" ) )  OR  ( TITLE-ABS-KEY ( "predictive model" ) ) )  OR  ( ( TITLE-ABS-KEY ( "clinical decision rule" ) )  OR  ( TITLE-ABS-KEY ( "clinical decision model" ) ) ) )  AND  ( TITLE-ABS-KEY ( retinopathy ) ) | 613 |
