# Supplementary material for: Prognostic models of diabetic microvascular complications: a systematic review and meta-analysis
Source: Syst Rev. 2021 Nov 1;10:288. doi: 10.1186/s13643-021-01841-z (PMC8561867; doi:10.1186/s13643-021-01841-z)
Supplement: Supplementary file 4 — Additional file 4: Figure S1. PRISMA diagram for diabetic retinopathy. Figure S2. PRISMA diagram for diabetic nephropathy. Figure S3. Risk-of-bias using PROBAST. Figure S4. Overview top 20 predictors included in derived models. Figure S5. Funnel plot (C-statistics) for DR in external validation. Figure S6. Funnel plot (C-statistics) for CKD in external validation. Figure S7. Funnel plot (C-statistics) for ESRD in external validation. [file 13643_2021_1841_MOESM4_ESM.docx]

## **Supplementary Table**

### **TABLE S1** Characteristics of studies included in the systematic review

| **Study** | **Country** | **Study setting** | **Data source** | **Study design** | **Sample size**  **(include**  **validation)** | **T2D diagnosis** | **Characteristics of T2D** | | | |
| --- | --- | --- | --- | --- | --- | --- | --- | --- | --- | --- |
|  |  |  |  |  |  |  | **Ethnicity** | **Mean age (years)** | **Male (%)** | **Duration (years)** |
| **Diabetic Retinopathy (n = 32)**  Conventional statistics (26); Machine learning (6) | | | | | | | | | | |
| 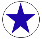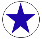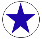 | | | | | | | | | | |
| Yusufi 2019[43] | India | Hospital | Medical records | CS | 388 | Medical records | Asian | 51.9 | 49.7 | 5.9 |
| Liao 2018[42] | Taiwan | Hospital | Medical records | GS | 1,597 | HbA1c ≥6.5/ FPG ≥7.0/ OGTT ≥11.1/ RPG ≥11.1 | Asian | 60.0 | 51.9 | 12.9 |
| Basu 2018[44] | USA & Canada | Community | Survey – Primary data | PC | 3,301 | FPG ≥7.0/ OGTT ≥11.1/ HbA1c ≥6.5/ RPG ≥11.1 | Mixed | 60.8 | 42.2 | - |
| Basu 2017[12] | USA & Canada | Hospital | Trial database | RCT | 10,654 | HbA1c ≥7.5, diabetic drug | Mixed | 62.8 | 61.9 | - |
| Cox 2015[41] | UK | Hospital | Medical records | PC | 803,044 | Medical records (ICD-X, Read code) | Mixed | 60.7 | 56.1 | - |
| Pedre 2015[46] | Spain | Hospital | Medical records | RC | 508 | Medical records | Caucasian | 61.5 | 60.8 | 12.0 |
| Heijden 2014[30] | Netherlands | Hospital | Medical records | PC | 3,319 | FPG ≥7.0/ OGTT ≥11.1/ RPG ≥11.1 | Caucasian | 60.5 | 54.2 | 1.4 |
| Oh E 2013[17] | Korea | Community | Survey data | CS | 490 | FPG ≥7.0, HbA1c ≥6.5/ RPG ≥11.1 | Asian | 60.8 | 51.6 | 6.2 |
| Wang 2014[47] | China | Hospital | Medical records | CS | 2,699 | FPG ≥7.0/ OGTT ≥11.1 | Asian | 58.8 | 46.8 | 5.6 |
| Aspelund 2011[11] | Denmark | Hospital | Medical records | PC | 5,199 | Medical records | Caucasian | - | - | - |
| Hosseini 2009[45] | Iran | Hospital | Medical records | CS | 3,734 | Medical records | Asian | 52.2 | - | 7.0 |
| 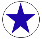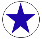 | | | | | | | | | | |
| Mo 2020[49] | China | Community | Survey – Primary data | CS | 4,170 | FPG ≥7.0/ OGTT ≥11.1/ RPG ≥11.1 | Asian | 64.5 | 42.2 | 9.7 |
| Zhu 2020[53] | China | Community | Survey – Primary data | CS | 421 | Medical records | Asian | 66.6 | 42.9 | 8.9 |
| Ochs 2019[50] | UK | Community | Medical records | RC | 220,276 | Medical records | - | 62.9 | 56.1 | 4.1 |
| Ogunyemi 2019[51] | USA | Hospital | Medical records | RC | 27,116 | Medical records | - | - | - | - |
| Tsao 2018[52] | Taiwan | Hospital | Medical records | CS | 536 | Medical records | Asian | 61.5 | 51.9 | 12.9 |
| Dagliati 2018[13] | Italy | Hospital | Medical records | RC | 1,000 | Medical records | Caucasian | - | - | - |
| Finana 2018[48] | UK | Hospital | Medical records | RC | 13,103 | Medical records | Mixed | 59.5 | 58.3 | 1.9 |
| Herrera 2017[57] | Mexico | Community | Survey – Primary data | PC | 1,000 | FPG ≥7.0/ RPG ≥11.1 | American | 57.2 | 27.0 | 7.5 |
| Soleiman 2015[54] | Iran | Hospital | Medical records | CS | 1,782 | FPG ≥7.0, OGTT ≥11.1 | Asian | 50.3 | 32.7 | 5.8 |
| Ogunyemi 2015[15] | USA | Hospital | Medical records | PC | 1,239 | FPG ≥7.0/ RPG ≥11.1/ HbA1c ≥6.5 | American | - | - | - |
| Ogunyemi 2013[16] | USA | Hospital | Medical records | RC | 513 | Medical records | American | - | - | - |
| Tanaka 2013[59] | Japan | Hospital | Trial database | RCT | 1,748 | HbA1c ≥7.0, diabetic drug | Asian | 62.1 | 50.1 | - |
| Semeraro 2011[18] | Italy | Hospital | Medical records | PC | 5,034 | FPG ≥7.0/ OGTT ≥11.1/ HbA1c ≥ 6.5/ RPG ≥11.1 | Caucasian | 61.0 | 60.0 | 2.0 |
| Ng 2008[58] | USA | Hospital | Medical records | PC | 18 | Medical records | American | 50.4 | 50.0 | 6.9 |
| Han 2004[56] | USA | Hospital | Medical records | PC | 28 | Medical records | American | 50.5 | 52.5 | 7.8 |
| 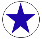 | | | | | | | | | | |
| Chong 2017[55] | Singapore | Community | Survey – Primary data | GS | 1,528 | RPG≥11.1/diabetic drug/ self-report | Asian | 62.5 | 51.3 | 10.2 |
| Mogilevskyy 2017[62] | Ukraine | Hospital | Medical records | GS | 409 | Medical records | Caucasian | - | - | - |
| Kengne 2015[14] | South Africa | Hospital | Medical records | CS | 213 | Medical records | African | 56.0 | 59.2 | 4.8 |
| Cichosz 2015[61] | USA | Community | Survey data | PC | 266 | OGTT ≥11.1, HbA1c ≥6.5/ FPG ≥7.0 | - | 63.8 | 54.9 | - |
| Welsh 2014[24] | UK | Hospital | Trial database | PC | 3,225 | Medical records | Caucasian | 66.6 | 59.8 | 7.7 |
| Bresnick 1987[60] | USA | Hospital | Medical records | PC | 85 | Diabetic drug/ insulin | American | 44.6 | 61.2 | 15.8 |
| **Chronic Kidney Disease (n = 30)**  Conventional statistics (26); Machine learning (4) | | | | | | | | | | |
| 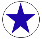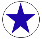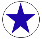 | | | | | | | | | | |
| Song 2019[71] | USA | Hospital | Medical records | RC | 15,645 | Diabetic drug/ HbA1c ≥6.5/ FPG ≥7.0/ RPG ≥11.1 | Mixed | 59.0 | 49.4 | - |
| Peters 2019[68] | Australia | Community | Medical records | RC | 792 | FPG ≥7.0 | Mixed | 65.6 | 54.3 | 7.0 |
| Nowak 2018[23] | USA | Hospital | Medical records | PC | 1,032 | Medical records | Mixed | 57.0 | 59.0 | 10.0 |
| Basu 2018[44] | USA – Canada | Community | Primary data collection | PC | 3,301 | FPG≥7.0/ OGTT≥11.1/ HbA1c ≥ 6.5/RPG≥11.1 | Mixed | 60.8 | 42.2 | - |
| Basu 2017[12] | USA – Canada | Hospital | Trial database | RCT | 9,635 | HbA1c ≥ 7.5, diabetic drug | Mixed | 62.8 | 61.9 | - |
| Blech 2011[19] | Israel | Hospital | Medical records | GS | 1,274 | Medical records | Asian | 61.9 | 46.9 | 20.2 |
| Wu 2017[25] | China | Hospital | Medical records – Primary data collection | PC | 4,795 | Medical records | Asian | 59.3 | 56.2 | 9.1 |
| Dunkler 2015[20] | Austria | Hospital | Trial database | PC | 6,766 | Medical records | Caucasian | 65.4 | 68.3 | 6.6 |
| Qian 2019[69] | Malaysia | Hospital | Medical records | RC | 377 | Medical records | Asian | 58.7 | 41.6 | - |
| Peters 2017[79] | Australia | Hospital | Medical records | PC | 345 | FPG >6.0, Medical records/ self-report | Mixed | 67.0 | 51.9 | 9.0 |
| 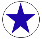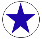 | | | | | | | | | | |
| Hu 2020[65] | China | Community | Medical records – Primary data collection | RC | 3,489 | FPG ≥7.0/ OGTT ≥11.1/ RPG ≥11.1 | Asian | 64.5 | 44.8 | 9.5 |
| Wysham 2020[72] | USA | Hospital | Medical records | RC | 160,031 | HbA1c ≥6.5/ FPG ≥7.0/ OGTT ≥11.1/ RPG ≥11.1 | Mixed | 66.5 | 53.4 | - |
| Jiang 2020[66] | China | Hospital | Medical records | RC | 302 | HbA1c ≥6.5, FPG ≥7.0/ OGTT ≥11.1 | Asian | 53 | 68.9 | 6.3 |
| Liao 2019[67] | Taiwan | Community | Medical records – Primary data collection | GS | 1,514 | Medical records (ICD IX: 250) | Asian | 62.9 | 50.8 | 9.7 |
| Romero 2019[70] | USA-Canada | Hospital | Medical records | RCT | 10,251 | FPG ≥7.0/ RPG ≥11.1/ OGTT ≥11.1 | Mixed | 63.0 | 61.0 | 10.0 |
| Dagliati 2018[13] | Italy | Hospital | Medical records | RC | 1,000 | Medical records | Caucasian | - | - | - |
| Miao 2017[22] | China | Community | Survey data | RC | 5,705 | FPG ≥7.0/ self-report | Asian | 55.4 | 40.6 | - |
| Low 2017[78] | Singapore | Hospital | Medical records – Primary data collection | PC | 1,107 | Medical records | Mixed | 57.3 | 57.9 | 10.0 |
| Tanaka 2013[59] | Japan | Hospital | Trial database | RCT | 1,748 | HbA1c ≥ 7.0 | Asian | 62.1 | 50.1 | - |
| Jardine 2012[74] | Australia | Hospital | Trial database | RCT | 7,377 | HbA1c ≥ 6.5/ self-report | Mixed | 66.0 | 57.2 | 7.0 |
| Goldfarb 2002[73] | USA | Community | Register | RC | 86 | FPG ≥7.0/ RPG ≥11.1/ OGTT≥11.1 | American | 44.0 | - | 13.0 |
| 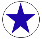 | | | | | | | | | | |
| Klisic 2018[21] | Montenegro | Hospital | Medical records – Primary data collection | CS | 106 | 2-FPG ≥7.0 /RPG ≥ 11.1/ HbA1c≥6.5/ self-report | - | 64.9 | 38.7 | - |
| Jenks 2017[75] | UK | Community | Medical records | PC | 1,066 | Diabetic drug, HbA1c ≥ 6.5 | Caucasian | 67.3 | 50.4 | 5.6 |
| Kim 2017[76] | Korea | Hospital | Medical records | RC | 325 | Medical records | - | 55.7 | 32.5 | 7.8 |
| Lindhart 2017[77] | UK | Hospital | Trial database | RCT | 737 | Medical records | Caucasian | 57.5 | 53.9 | 8.9 |
| Saulnier 2017[81] | France | Hospital | Medical records | PC | 1,135 | Medical records | Caucasian | 64.0 | 57.4 | 14.0 |
| Yang 2017[82] | China | Hospital | Medical records | PC | 210 | HbA1c ≥ 7.5% | - | 64.3 | 51.9 | 6.6 |
| Zobel 2017[83] | Denmark | Hospital | Medical records | PC | 200 | FPG ≥7.0/ OGTT ≥11.1/ HbA1c ≥6.5/ RPG ≥11.1 | - | 59.0 | 76.0 | 13.0 |
| Riphagen 2015[80] | Netherlands | Hospital | Medical records | PC | 640 | 2-FPG ≥7.0, FPG ≥7.0/ OGTT ≥11.1 | - | 66.0 | 39.4 | 5.0 |
| Welsh 2014[24] | UK | Hospital | Trial database | PC | 3,385 | HbA1c ≥ 6.5 | Caucasian | 66.6 | 59.8 | 7.7 |
| **End Stage Renal Disease (n = 18)**  Conventional statistics (18); Machine learning (0) | | | | | | | | | | |
| 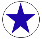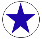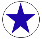 | | | | | | | | | | |
| Basu 2018[44] | USA | Community | Survey data | PC | 3,301 | FPG ≥7.0/ OGTT ≥11.1/ HbA1c ≥6.5/RPG ≥11.1 | Mixed | 60.8 | 42.2 | - |
| Basu 2017[12] | USA | Hospital | Trial database | RCT | 9,635 | HbA1c ≥ 7.5, diabetic drug | Mixed | 62.8 | 61.9 | - |
| Wan 2017[29] | Hong Kong | Hospital | Medical records | RC | 116,509 | Medical records | Asian | 62.1 | 46.7 | 7.1 |
| Elley 2013[26] | New Zealand | Hospital | Medical records | PC | 31,613 | Medical records | Mixed | 60.8 | 49.5 | 4.4 |
| 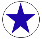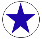 | | | | | | | | | | |
| Wysham 2020[72] | USA | Hospital | Medical records | RC | 160,031 | HbA1c ≥6.5/ FPG ≥7.0/ OGTT ≥11.1/ RPG ≥11.1 | Mixed | 66.5 | 53.4 | - |
| Lin 2017[84] | Taiwan | Hospital | Medical records | RC | 24,104 | Medical records, HbA1c ≥ 6.0 | Asian | 61.0 | 46.8 | 6.7 |
| Li 2016[28] | Taiwan | Hospital | Medical records | PC | 604 | Medical records, HbA1c ≥ 6.0 | - | 61.7 | 48.7 | 8.2 |
| Jardine 2012[74] | Australia | Hospital | Trial database | RCT | 10,506 | HbA1c ≥ 6.5, self-report | Mixed | 66.0 | 57.5 | 7.0 |
| Yang 2006[85] | Hong Kong | Community | Medical records | PC | 4,438 | Medical records | Asian | 60.3 | 42.7 | 6.2 |
| 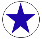 | | | | | | | | | | |
| Garlo 2018[27] | USA | Hospital | Trial database | RCT | 5,367 | HbA1c ≥ 6.5/ diabetic drugs (insulin) | Mixed | 61.0 | 67.9 | 9.1 |
| Mise 2017[90] | Japan | Hospital | Medical records | RC | 136 | Medical records, HbA1c ≥ 6.0 | Asian | 61.0 | 80.2 | 14.0 |
| Mise 2016[89] | Japan | Hospital | Medical records | RC | 149 | Medical records | - | 61.0 | 79.2 | 14.0 |
| Foster 2015[86] | USA | Community | Medical records | PC | 250 | Medical records, OGTT ≥ 11.1 | American | 42.4 | 30.8 | 11.2 |
| Fufaa 2015[87] | USA | Community | Medical records | PC | 260 | Medical records, OGTT ≥ 11.1 | American | 42.5 | 31.5 | 11.4 |
| Pavkov 2013[92] | India | Community | Medical records | PC | 234 | Medical records, OGTT ≥ 11.1 | American | 42.8 | 33.8 | 12.3 |
| Niewczas 2012[91] | USA | Hospital | Medical records | PC | 410 | Medical records | Mixed | 55.9 | 56.0 | 13.5 |
| Heersprink 2010[88] | Netherlands | Hospital | Trial database | RCT | 701 | Medical records | Mixed | 60.4 | 62.2 | - |
| Keane 2006[93] | Multi country | Hospital | Trial databases | RCT | 1,513 | Oral diabetic drug, insulin use, no ketoacidosis | Mixed | 59.6 | 63.2 | - |

### **TABLE S1** Continued

| **Study** | **Complications** | | | | | **Prognostic models** | | | | |
| --- | --- | --- | --- | --- | --- | --- | --- | --- | --- | --- |
|  | **Definition & ascertainment** | **Prevalence at baseline (%)** | **Follow up (years)** | **LFU**  **(%)** | **Incidence (%)** | **Handling**  **Missing data (%)** | **Phase** | **Internal validation** | **Statistics** | **Predicted horizon (years)** |
| **Diabetic Retinopathy (n = 32)**  Conventional statistics (26); Machine learning (6) | | | | | | | | | | |
| 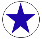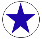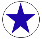 | | | | | | | | | | |
| Yusufi 2019 [43] | Fundus examination | 14.2 | - | - | - | Mean imputation | E | - | Logit | 1,2,3 |
| Liao 2018[42] | Fundus examination | 35.2 | - | - | - | Genotype input | D/I/E | SS, CV | Logit | - |
| Basu 2018[44] | Retinal photocoagulation | - | 9.1/8.0 | - | 2.2/5.4 | MICE (~9.1) | E | - | Cox | 10 |
| Basu 2017[12] | Retinal photocoagulation | - | 4.7 | - | 15.2 | MICE (~6.0) | D/I/E | CV | Cox | 10 |
| Cox 2015 [41] | Retinal photocoagulation | - | - | - | 1.69 | MI (-) | D/I/E | SS | Cox | 10 |
| Pedre 2015[46] | Fundus examination | 32.7 | 1.1 | - | 3.1 | - | E | - | Cox | - |
| Heijden 2014[30] | Fundus examination | 10.2 | 4.4 | 16.3 | 2.3 | CD (~18.8) | E | - | Poisson | - |
| Oh E 2013[17] | Fundus examination | 17.1 | - |  | - | - | D/I/E | SS, CV | Logit, ML | - |
| Wang 2014[47] | Fundus examination | 19.7 | - | - | - | - | D/I | CV | Logit | - |
| Aspelund 2011[11] | Fundus examination | - | 20.0 | - | 5.2 | MI (22.0-67.0) | D | - | Weibull survival | - |
| Hosseini 2009[45] | Fundus examination | 54.0 | - | - | - | CD (-) | D | - | Logit | - |
| 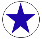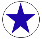 | | | | | | | | | | |
| Mo 2020[49] | Fundus examination | 21.3 | - | - | - | - | D/I | SS | Logit | - |
| Zhu 2020[53] | Fundus examination | 42.9 | - | - | - | - | D/I | SS | Logit | - |
| Ochs 2019[50] | Retinal photocoagulation | - | 10 | 7.7 | 42 | MICE (-) | D/I | SS | Poisson, GLM | - |
| Ogunyemi 2019[51] | Fundus examination | - | - | - | 34 | k-NN | D/I | SS, CV | Logit, SVM, NN | - |
| Tsao 2018[52] | Fundus examination | 19.8 | - | - | - | - | D/I | SS, LOO | Logit, SVM, NN, DT | 2,5,10 |
| Dagliati 2018[13] | Fundus examination | - | 4.0 | - | 12.5 | RF (~36.2) | D/I | LOO | Logit, DT, NB, SVM | 3,5,7 |
| Finana 2018[48] | Retinal photocoagulation | 0.2 | 6.2 | - | 1.52 | CD (~36) | D/I | SS | GLM | 1 |
| Herrera 2017[57] | Ocular assessment | - | 3.0 | - | 31.7 | MICE (~5) | D/I | SS, CV | Probit | - |
| Soleiman 2015[54] | Ocular assessment | 57.2 | - | - | - | CD (-) | D/I | SS | Logit | - |
| Ogunyemi 2015[15] | Retinal photocoagulation | - | 4.0 | - | 12.8 | MI (~50.0) | D/I | CV | AB, RB | - |
| Ogunyemi 2013[16] | Retinal photocoagulation | - | - | - | 25.3 | - | D/I | CV | BN, NN | - |
| Tanaka 2013[59] | Ocular assessment | - | 7.2 | - | 30.2 | MI (-) | D/I | CV | Cox | 5 |
| Semeraro 2011[18] | Fundus examination | - | 11 | 31.3 | 20.7 | - | D/I | SS | Cox | 1,2,3,4 |
| Ng 2008[58] | Fundus examination | - | 3.0 | 28.6 | 16.7 | - | D/I | CV | Logit | 3 |
| Han 2004[56] | Fundus examination | - | 1.0 | - | 42.9 | - | D/I | SS | Logit | 1 |
| 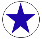 | | | | | | | | | | |
| Chong 2017[55] | Retinal photocoagulation | 35.8 | - | - | - | MI (-) | D | - | Logit | - |
| Mogilevskyy 2017[62] | Fundus examination | 31.3 | - | - | - | - | D | - | Logit | - |
| Kengne 2015[14] | Fundus examination | 25.4 | - | - | - | - | D | - | Logit | 10 |
| Cichosz 2015[61] | Fundus examination | - | 4.0 | - | 16.5 | MI (1.1-14.0) | D | - | Logit | - |
| Welsh 2014[24] | Retinal photocoagulation | - | 5.0 | 2.4 | 5.7 | - | D | - | Cox | 5 |
| Bresnick 1987[60] | Fundus examination | - | 5.0 | - | 30.6 | - | D | - | Cox | 2 |
| **Chronic Kidney Disease (n = 30)**  Conventional statistics (26); Machine learning (4) | | | | | | | | | | |
| 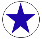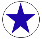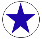 | | | | | | | | | | |
| Song 2019[71] | ACR ≥30/ UPCR ≥30/ MDRD eGFR <60 | 27.1 | 10.0 | - | 35.7 | Gradient boosting | D/I/E | SS, BTR | Gradient boosting | - |
| Peters 2019[68] | EPI eGFR <60 | - | 4.2 | - | 11.2 | - | D/I/E | BTR | Logit | 4 |
| Nowak 2018[23] | EPI eGFR declined ≥ 30% | 41.7 | 7.9 | - | 11.1 | - | D/I/E | CV | Logit | 5 |
| Basu 2018[44] | UACR: ≥30, ≥300, dialysis/ S-Cr>3.3 | - | 9.1/8.0 | - | 18.7/10.1 | MICE (~9.1) | E | - | Cox | - |
| Basu 2017[12] | UACR: <30, 30-300, >300 | - | 4.7 | - | 24.1 | MICE (~6.0) | D/I/E | CV | Cox | 10 |
| Blech 2011[19] | Creatinine: 0.03-0.3, >0.3/ dialysis | 38.9 | - | - | - | - | D/I/E | SS | Logit | - |
| Wu 2017[25] | UAE: <30, 30-300, ≥300 | - | 6.0 | 10.6 | 12.3 | CD (-) | D/E | - | Logit | 6 |
| Dunkler 2015[20] | UACR: 30-299, ≥300 | - | 5.5 | - | 15.9/14.9 | CD (0.2-5.0) | D/E | - | Logit | 5.5 |
| Qian 2019[69] | GFR <45, proteinuria, dialysis, RRT | - | 5.0 | - | - | CD | E | - | Logit | - |
| Peters 2017[79] | EPI eGFR<60/; eGFR declined ≥30%; eGFR declined ≥ 5mL/year; UACR: ≥3, ≥30 | 13.0 | 4.0 | - | 10.1 | - | D/I | BTR | Logit | 4 |
| 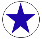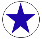 | | | | | | | | | | |
| Hu 2020[65] | UACR: <30, 30-300, >300 | - | - | - | 20.1 | CD (-) | D/I | BTR | Logit | - |
| Wysham 2020[72] | eGFR <60 | - | - | 10.0 | 6.9 | - | D/I | SS | Logit | - |
| Jiang 2020[66] | eGFR <60, kidney damage >3 months | - | 5.5 | 1.2 | 47.4 | CD | D/I | SS | Logit | - |
| Liao 2019[67] | MDRD eGFR <60, dipstick proteinuria >1 | 28.1 | - | - | - | - | D/I | SS | Logit | - |
| Romero 2019[70] | 2S-Cr >20/ UACR: ≥30, ≥300/ dialysis/ RRT | - | 7.0 | - | 66.0 | - | D/I | SS, BTR | RF, Logit, NB, DT | - |
| Dagliati 2018[13] | eGFR: < 60/ ACR: 30-299 | - | 4.0 | - | 12.8 | RF (0.01-36.2) | D/I | LOO | Logit, DT, SVM, NB | 3,5,7 |
| Miao 2017[22] | AER: 30-300, >300 | - | - | - | 0.7 | MI (-) | D/I | - | Cox | 5,10,20 |
| Low 2017[78] | MDRD eGFR < 60 | - | 5.6 | - | 42.2 | - | D/I | SS | Logit | 6 |
| Tanaka 2013[59] | UAE > 33.9 | - | 7.2 | - | 4.1 | MI (-) | D/I | CV | Cox | 5 |
| Jardine 2012[74] | 2-SCr ≥ 2.26/ ACR ≥ 30 | - | 5.0 | 14.9 | 37.3 | - | D/I | BTR | Cox | 5 |
| Goldfarb 2002[73] | ACR > 30 | - | 4.0 | - | 46.5 | CD (-) | D/I | - | Logit, NN, DT, GA | 4 |
| 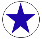 | | | | | | | | | | |
| Klisic 2018[21] | MDRD eGFR <60 | 37.7 | - | - | - | - | D | - | Logit | - |
| Jenks 2017[75] | EPI eGFR :<60, declined >25% | 33 | 6.7 | - | 28.4 | - | D | - | Cox | 6.7 |
| Kim 2017[76] | EPI eGFR: <15,15-29,30-59,60-90,>90 | 37.5 | 4.3 | 35.7 | 7.7 | - | D | - | Cox | 5 |
| Lindhart 2017[77] | 3-UAE: <20, ≥20 µg/L) | - | 4.7 | 9.8 | 12.1 | - | D | - | Cox | - |
| Saulnier 2017[81] | EPI eGFR declined ≥40%, declined ≤5 | - | 4.6 | 25.7 | 35.0 | - | D | - | Cox | 5 |
| Yang 2017[82] | MDRD eGFR < 60 | - | 5.3 | - | 47.6 | - | D | - | Logit | 5.3 |
| Zobel 2017[83] | EPI eGFR declined > 30% | - | 6.2 | - | 21.0 | - | D | - | Cox | 6.1 |
| Riphagen 2015[80] | ACR: ≥2.5 (M), ≥3.5 (F); 50% increase 2-SCr | 40 | 10.0 | - | 28.6 | MICE (-) | D | - | Cox | 10 |
| Welsh 2014[24] | ACR: ≥ 300/ 2-SCr ≥ 200 | - | 5.0 | 3.9 | 8.8 | - | D | - | Cox | 5 |
| **End Stage Renal Disease (ESRD)**  Conventional statistics (18); Machine learning (0) | | | | | | | | | | |
| 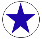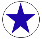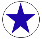 | | | | | | | | | | |
| Basu 2017[12] | Dialysis/ S-Cr >3.3, 2 S-Cr >20 | - | 4.7 | - | 3.0 | MICE (~6.0) | D/I/E | CV | Cox | 10 |
| Basu 2018[44] | Dialysis/ S-Cr >3.3, 2 S-Cr >20 | - | 9.1/8.0 | - | 25.5 | MICE (~9.1) | E | - | Cox | 10 |
| Wan 2017[29] | ICD-IX (250.3,585,586)/ eGFR <15 | - | 4.1 | 2.8 | 41.5 | MICE (-) | D/E | SS | Cox | 5 |
| Elley 2013[26] | Dialysis, RRT, renal failure | - | 7.3 | - | 2.5 | CD (~40) | D/E | - | Cox | 5 |
| 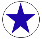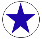 | | | | | | | | | | |
| Wysham 2020[72] | eGFR <15, dialysis, RRT, renal death | - | 10.0 | - | 1.4/1.9 | - | D/I | SS | Logit | - |
| Lin 2017[84] | ICD-IX, 2 S-Cr > 2.26, RRT, renal death | 0.9 | 8.3 | - | 5.0 | MI (-) | D/I | BTR | Cox | 3,5,8 |
| Li 2016[28] | Dialysis/ EPI eGFR <15 | - | 12.6 | - | 3.6 | - | D/I | LOO | Cox | 5,10,15 |
| Jardine 2012[74] | 2-S-Cr ≥ 2.26, RRT/ renal failure | - | 4.8 | 14.9 | 2.4 | CD (-) | D/I | BTR | Cox | 5 |
| Yang 2006[85] | ICD-IX (250.4, 585, 586), non-fatal renal failure (585, 586)/ eGFR <15 | - | 2.9 | - | 3.2 | CD (-) | D/I | SS | Cox | 4 |
| 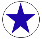 | | | | | | | | | | |
| Garlo 2018[27] | Dialysis/ EPI eGFR <15 | 1.7 | 1.5 | - | 1.8 | MI (~25.0) | D | - | Cox | 1 |
| Mise 2017[90] | Dialysis/ EPI eGFR declined ≥ 40% | - | 2.0 | - | 76.5 | - | D | - | Cox | 2 |
| Mise 2016[89] | Dialysis/ EPI eGFR declined ≥ 40% | 2.6 | 2.8 | - | 63.1 | - | D | - | Cox | 10 |
| Foster 2015[86] | RRT/ Renal failure death / ICD-IX (250.4) | - | 14* | - | 27.6 | CD (-) | D | - | Cox | 10 |
| Fufaa 2015[87] | RRT/ Renal failure death | - | 13 | - | 28.5 | - | D | - | Cox | 13 |
| Pavkov 2013[92] | Dialysis, RRT/ renal failure | - | 10.8 | - | 29.1 | - | D | - | Cox | - |
| Niewczas 2012[91] | MDRD eGFR <15, dialysis, RRT, death | - | 12 | - | 14.4 | - | D | - | Cox | 12 |
| Heersprink 2010[88] | Dialysis, RRT, renal death | - | 3.4 | - | 28.8 | - | D | - | Cox | - |
| Keane 2006[93] | Dialysis, RRT | - | 4.5 | - | 22.2 | - | D | - | Cox | - |

**Abbreviations**: AB, adaptive boosting; ACR, albumin-to-creatinine ratio (mg/mmol or mg/g); AER, Albumin excretion rate; BN, Bayesian network; BTR, bootstrapping; CD, case deletion; CKD, chronic kidney disease; CKD-EPI, chronic kidney disease epidemiology collaboration; CKD-MDRD, chronic kidney disease modification of diet in renal disease study; CS, cross sectional; CV, cross validation; D, derivative; DT, decision tree; E, external validation; eGFR, Estimated Glomerular Filtration Rate (ml/min/1.73 m^2^); ESRD, End Stage Renal Disease; FPG, fasting plasma glucose (mmol/L); GA, generalised additive; GLM, Generalized Linear model; GS, Genetic Studies; HbA1c, glycated haemoglobin (%); I, internal validation; ICD, International Statistical Classification of Diseases; k-NN, k-nearest neighbour; LFU, loss to follow up; LOO, leave one out; MI, multiple imputation; MICE, multiple imputation by chained equations; NB, naïve Bayesian; NN, neural network; OGTT, oral glucose tolerance test (mmol/L); PC, Prospective cohort; RB, Random under sampling boost; RC, Retrospective cohort; RCT, randomised controlled trial; RF, random forest; RPG, Random plasma glucose (mmol/L); RRT, Renal replacement therapy, S-Cr, Serum creatinine; SS, split sample; SVM, support vector machine; UACR, urine albumin creatinine ratio (mg/g); UAE, urine albumin excretion (mg/24-h)

### **TABLE S2** Describe discrimination and calibration performances of prognostic models

| **Study** | **Model** | **Derivative model**  **(N_1_/N_2_/N_3_)** | | | | **Internal Validation**  **(N_1_/N_2_/N_3_)** | | | **Author**  **(Year)** | **External Validation**  **(N_1_/N_2_/N_3_)** | | |
| --- | --- | --- | --- | --- | --- | --- | --- | --- | --- | --- | --- | --- |
|  |  | **No. Variable/ event/n** | **Calibration** | **Discrimination**  **(C-statistics)** | **No. Variable/ event/n** | | **Calibration** | **Discrimination**  **(C-statistics)** |  | **No. Variable/ event/n** | **Calibration** | **Discrimination**  **(C-statistics)** |
| **Diabetic Retinopathy (n = 32)** | | | | | | | | | | | | |
|  | | **28/46/38** | | | **20/30/35** | | | |  | **8/19/5** | | |
| 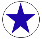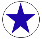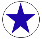 | | | | | | | | | | | | |
| Liao 2018[42] | Logit  Logit | 5/234/528  4/234/528 | -  - | 0.77(0.73-0.81)  0.72(0.67-0.76) | 5/234/528  4/234/528 | | -  - | 0.74(0.70-0.79)  0.73(0.69-0.77) | Liao 2018[42] | 5/95/542  4/95/542 | -  - | 0.66(0.60-0.72)  0.65(0.59-0.72) |
| Basu 2017[12] | Cox  Cox  Cox  Cox  Cox | 12/901/9635  12/1476/9635  12/3559/9635  12/776/9635  12/1468/9635 | Fair  Fair  Fair  Fair  Fair | -  -  -  0.57(0.55-0.58)  - | 12/901/9635  12/1476/9635  12/3559/9635  12/776/9635  12/1468/9635 | | 1.03;15.7(0.07)  0.97;18.7(0.03)  0.78;9.2(0.42)  1.01;6.9(0.65)  0.97;11.5(0.24) | 0.65(0.63-0.67)  0.68(0.66.0.69)  0.55(0.54-0.56)  0.62(0.60-0.64)  0.63(0.62-0.65) | Basu 2017[12] | 12/115/1018 | 0.72;13.9(0.05) | 0.57(0.51-0.63) |
|  |  |  |  |  |  |  |  |  |  | -  -  -  - | -  -  1.12;59.0(0.01)  - | -  -  0.59(0.57-0.62)  - |
|  |  |  |  |  |  | |  |  | Basu 2018[44] | 11/34/1555  11/94/1746 | 1.07;7.70(0.05)  0.47;10.9(0.14) | 0.76(0.67-0.86)  0.64(0.58-0.70) |
| Oh 2013[17] | Lasso LR | 12/56/ 327 | - | 0.82 (0.75-0.89) | 12/ - /163 | | - | 0.81(0.74-0.86) | Oh 2013[17] | 12/144/562 | - | 0.82(0.78-0.85) |
|  | Ridge LR | 18/56/ 327 | - | 0.81 (0.74-0.88) | - | | - | - | - | - | - | - |
|  | Elastic LR | 16/56/ 327 | - | 0.80 (0.82-0.94) | - | | - | - | - | - | - | - |
|  | Logit BS | 10/56/ 327 | - | - | 10/ - /163 | | - | 0.79(0.72-0.85) |  | 10/ - /562 | - | 0.79(0.75-0.83) |
|  | SVM | 37/56/ 327 | - | - | 37/ - /163 | | - | 0.83(0.76-0.88) |  | 37/ - /562 | - | 0.81(0.78-0.84) |
|  | NN | 37/56/ 327 | - | - | 37/ - /163 | | - | 0.79(0.72-0.85) |  | 37/ - /562 | - | 0.79(0.76-0.83) |
|  | DT | 37/56/ 327 | - | - | 37/ - /163 | | - | 0.80(0.73-0.85) |  | 37/ - /562 | - | 0.76(0.72-0.79) |
|  | NB | 37/56/ 327 | - | - | 37/ - /163 | | - | 0.76(0.69-0.82) |  | 37/ - /562 | - | 0.73(0.69-0.77) |
|  | K-NN | 37/56/ 327 | - | - | 37/ - /163 | | - | 0.52(0.45-0.59) |  | 37/ - /562 | - | 0.52(0.48-0.57) |
| Cox 2015[41] | Cox  Cox | 9/3989/254896  9/4074/199679 | - | - | 9/1286/80012  9/1365/62407 | | Fair  Fair | 0.75(0.73-0.77)  0.73(0.71-0.74)  0.74(0.74-0.74) | Cox 2015[41] | 9/1358/115770  9/1487//90280 | Fair  Fair | 0.77(0.75-0.78)  0.73(0.72-0.75)  0.75(0.75-0.75) |
| Aspelund 2011[11] | Weibull | 6/270/ 5199 | Fair | 0.76(0.74-0.78) | - | | - | - | Pedre 2015[46] | 6/166/508 | 0.87(0.80-0.94) | 0.74(0.62-0.85) |
|  |  |  |  |  |  | |  |  | Heijden 2014[30] | 6/415/3319 | 1.00(0.99-1.01) | 0.83(0.74-0.92) |
| Wang 2014[47] | Logit | 4/364/1869 | - | 0.70(0.67-0.73) | 4/167/830 | | - | 0.63(0.58-0.68) | Yusufi 2019[43] | 4/23/284  4/23/284  4/32/104  4/21/93  4/12/84 | -  -  -  -  - | 0.78(0.73-0.83)  0.56(0.51-0.63)  0.82(0.73-0.89)  0.81(0.72-0.88)  0.69(0.58-0.78) |
| Hosseini 2009[45] | Logit | 5/2017/3734 | - | 0.70(0.68-0.72) | - | | - | - | Yusufi 2019[43] | 5/23/284  5/23/284 | -  - | 0.82(0.76-0.86)  0.62(0.56-0.68) |
| 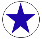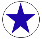 |  |  |  |  |  | |  |  |  |  |  |  |
| Mo 2020[49] | Logit | 7/667/3130 | Fair; O/E=1.00 | 0.70(0.68-0.72) | 7/222/1040 | | Fair; O/E=1.10 | 0.72(0.67-0.76) | - | - | - | - |
| Zhu 2020[53] | Logit | 5/69/336 | - | 0.88(0.83-0.94) | 5/18/85 | | - | 0.80(0.67-0.93) | - | - | - | - |
| Ochs 2019[50] | GLM | 9/ - /142264 | - | 0.79(0.78-0.81) | 9/ - /60970 | | - | - | - | - | - | - |
| Ogunyemi 2019[51] | Logit  SVM  NN | 8/6155/18077  8/6155/18077  8/6155/18077 | -  -  - | 0.76(0.75-0.77)  0.75(0.74-0.76)  0.75(0.74-0.76) | 8/3078/9039  8/3078/9039  8/3078/9039 | | -  -  - | 0.75(0.74-0.76)  0.74(0.73-0.75)  0.75(0.74-0.76) | -  -  - | -  -  - | -  -  - | -  -  - |
|  | Logit  SVM  NN | 8/6155/18077  8/6155/18077  8/6155/18077 | -  -  - | 0.75(0.74-0.76)  0.72(0.71-0.73)  0.76(0.75-0.77) | 8/3078/9039  8/3078/9039  8/3078/9039 | | -  -  - | 0.75(0.74-0.76)  0.72(0.70-0.73)  0.76(0.75-0.77) | -  -  - | -  -  - | -  -  - | -  -  - |
| Tsao 2018[52]  (Splitting 80:20) | Logit  SVM  DT  NN | 10/85/428  10/85/428  10/85/428  10/85/428 | -  -  -  - | 0.75(0.69-0.81)  0.78(0.72-0.84)  0.72(0.65-0.79)  0.88(0.83-0.92) | 10/21/108  10/21/108  10/21/108  10/21/108 | | -  -  -  - | 0.80(0.68-0.92)  0.84(0.73-0.95)  0.77(0.64-0.89)  0.77(0.65-0.90) | -  -  -  - | -  -  -  - | -  -  -  - | -  -  -  - |
| Tsao 2018[52]  (Splitting 60:20:20) | Logit  SVM  DT  NN  Logit  SVM  DT  NN | 10/64/322  10/64/322  10/64/322  10/64/322  -  -  -  - | -  -  -  -  -  -  -  - | 0.77(0.69-0.84)  0.96(0.93-0.99)  0.71(0.63-0.78)  0.85(0.79-0.91)  -  -  -  - | 10/21/108  10/21/108  10/21/108  10/21/108  10/21/108  10/21/108  10/21/108  10/21/108 | | -  -  -  -  -  -  -  - | 0.71(0.64-0.79)  0.74(0.67-0.82)  0.64(0.56-0.72)  0.69(0.61-0.76)  0.71(0.57-0.84)  0.80(0.58-0.92)  0.69(0.56-0.83)  0.70(0.57-0.84) | -  -  -  -  -  -  -  - | -  -  -  -  -  -  -  - | -  -  -  -  -  -  -  - | -  -  -  -  -  -  -  - |
|  | Logit  SVM  DT  NN | 10/106/536  10/106/536  10/106/536  10/106/536 | -  -  -  - | 0.76(0.69-0.81)  0.82(0.77-0.87)  0.69(0.63-0.75)  0.74(0.68-0.79) | 10/106/536  10/106/536  10/106/536  10/106/536 | | -  -  -  - | -  -  -  - | -  -  -  - | -  -  -  - | -  -  -  - | -  -  -  - |
|  | DT 2y  DT 5y  DT 10y | 10/106/536  10/106/536  10/106/536 | -  -  - | 0.50(0.36-0.64)  0.66(0.52-0.79)  0.70(0.57-0.84) | 10/106/536  10/106/536  10/106/536 | | -  -  - | -  -  - | -  -  - | -  -  - | -  -  - | -  -  - |
| Dagliati 2018[13] | Logit 3y  Logit 5y  Logit 7y | 5/125/ 1000  5/125/ 1000  5/125/ 1000 | -  -  - | 0.81(0.77-0.85)  0.77(0.72-0.82)  0.73(0.67-0.78) | 5/125/ 1000  5/125/ 1000  5/125/ 1000 | | -  -  - | 0.81(0.77-0.85)  0.77(0.72-0.82)  0.73(0.67-0.78) | -  -  - | -  -  - | -  -  - | -  -  - |
|  | RF 3y  RF 5y  RF 7y | 5/125/ 1000  5/125/ 1000  5/125/ 1000 | -  -  - | 0.85(0.82-0.88)  0.82(0.78-0.86)  0.80(0.76-0.85) | 5/125/ 1000  5/125/ 1000  5/125/ 1000 | | -  - | 0.85(0.82-0.88)  0.82(0.78-0.86)  0.80(0.76-0.85) | -  -  - | -  -  - | -  -  - | -  -  - |
|  | SVM 3y  SVM 5y  SVM 7y | 5/125/ 1000  5/125/ 1000  5/125/ 1000 | -  -  - | 0.82(0.79-0.85)  0.78(0.73-0.82)  0.74(0.69-0.79) | 5/125/ 1000  5/125/ 1000  5/125/ 1000 | | - | 0.82(0.79-0.85)  0.78(0.73-0.82)  0.74(0.69-0.79) | -  -  - | -  -  - | -  -  - | -  -  - |
|  | NB 3y  NB 5y  NB 7y | 5/125/ 1000  5/125/ 1000  5/125/ 1000 | -  -  - | 0.55(0.53-0.58)  0.56(0.53-0.58)  0.55(0.52-0.59) | 5/125/ 1000  5/125/ 1000  5/125/ 1000 | | - | 0.55(0.53-0.58)  0.56(0.53-0.58)  0.55(0.52-0.59) | -  -  - | -  -  - | -  -  - | -  -  - |
| Finana 2018[48] | GLM | 4/238/ 9172 | - | 0.90(0.86-0.92) | 4/103/ 3931 | | - | - | - | - | - | - |
| Herrera 2017[57] | Probit | 4/317/1000 | - | 0.78(0.74-0.82) | 4/32/100 | | - | 0.78(0.67-0.88) | - | - | - | - |
| Soleiman 2015[54] | Logit | 9/644/1143 | HL (p=0.65) | - | 9/161/286 | | 1.02(1.01-1.03) | 0.76(0.71-0.81) | - | - | - | - |
| Ogunyemi 2015[15] | RB  RB | 33/126/991  11/126/991 | -  - | 0.62(0.56-0.68)  0.59(0.53-0.65) | 33/32/248  11/32/248 | | - | 0.71(0.60-0.82)  0.72(0.62-0.82) | -  - | -  - | -  - | -  - |
|  | AB  AB | 33/126/991  11/126/991 | -  - | 0.55(0.50-0.60)  0.53(0.48-0.58) | 33/32/248  11/32/248 | | - | 0.60(0.49-0.71)  0.60(0.49-0.71) | -  - | -  - | -  - | -  - |
| Ogunyemi 2013[16] | BN | 23/130/513 | - | 0.60(0.54-0.66) | 23/130/513 | | - | - | - | - | - | - |
|  | NN | 23/130/513 | - | 0.54(0.48-0.60) | 23/130/513 | | - | - | - | - | - | - |
|  | BN | 6/130/513 | - | 0.60(0.54-0.66) | 6/130/513 | | - | - | - | - | - | - |
|  | NN | 6/130/513 | - | 0.56(0.50-0.62) | 6/130/513 | | - | - | - | - | - | - |
| Tanaka 2013[59] | Cox | 5/415/1748 | HL(p=0.13) | 0.61(0.52-0.70) | 5/415/1748 | | HL (p=0.13) | 0.61(0.52-0.70) | - | - | - | - |
| Semeraro 2011[18] | Cox | 7/376/3327 | Fair;0.68(0.20) | 0.75(0.72-0.78) | 7/189/1707 | | Fair;0.69(0.34) | 0.77(0.73-0.81) | - | - | - | - |
| Ng 2008[58] | Logit | 3/3/18 | - | 0.95(0.94-0.96) | 3/3/18 | | - | 0.92(0.88-0.96) | - | - | - | - |
| Han 2004[56] | Logit | 4/12/28 | - | 0.90(0.88-0.92) | 4/5/12 | | - | 0.88(0.83-0.92) | - | - | - | - |
| 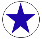 |  |  |  |  |  | |  |  |  |  |  |  |
| Chong 2017[55] | Logit  Logit  Logit  Logit | 7/547/1528  7/547/1528  6/547/1528  6/547/1528 | -  -  -  - | 0.74(0.71-0.76)  0.73(0.70-0.76)  0.71(0.68-0.74)  0.71(0.68-0.74) | -  -  -  - | | -  -  -  - | -  -  -  - | -  -  -  - | -  -  -  - | -  -  -  - | -  -  -  - |
| Mogilevskyy 2017[62] | Logit | 2/128/409 | - | 0.70(0.62-0.76) | - | | - | - | - | - | - | - |
| Kengne 2015[14] | Logit | 7/54/ 213 | Fair;5.9(0.66) | 0.88(0.82-0.93) | - | | - | - | - | - | - | - |
| Cichosz 2015[61] | Logit | 7/44/266 | Fair | 0.74(0.65-0.83) | - | | - | - | - | - | - | - |
| Welsh 2014[24] | Cox  Cox  Cox  Cox | 18/147/3225  17/147/3225  17/147/3225  16/147/3225 | -  -  -  - | 0.73(0.69-0.78)  0.73(0.68-0.77)  0.72(0.68-0.77)  0.72(0.68-0.76) | -  -  -  - | | -  -  -  - | -  -  -  - | -  -  -  - | -  -  -  - | -  -  -  - | -  -  -  - |
| Bresnick 1987[60] | Cox | 6/26/85 | - | - | - | | - | - | - | - | - | - |
| **Chronic Kidney Disease (n = 30)** | | | | | | | | | | | | |
|  |  | **28/79/17** | | | **17/40/17** | | | |  | **9/18/1** | | |
| 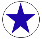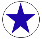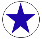 | | | | | | | | | | | | |
| Song 2019[71] | GBM | 440/3596/8098 | - | 0.82(0.81-0.83) | 440/1557/3461 | | - | 0.82(0.81-0.83) | Song 2019[71] | 400/412/4086 | - | 0.71(0.68-0.73) |
| Peters 2019[68] | Logit  Logit  Logit | 6/37/290  6/30/333  6/28/333 | 0.84;8.50(0.39)  1.00;14.7(0.06)  1.02;14.3(0.07) | 0.89(0.85-0.94)  0.81(0.75-0.87)  0.70(0.61-0.80) | 6/37/290  6/30/333  6/28/333 | | -  -  - | 0.87(0.79-0.95)  0.77(0.67-0.87)  0.64(0.53-0.76) | Peters 2019[68] | 6/36/362  6/24/413  6/31/413 | 1.07; 5.6 (0.06)  1.09;7.10(0.62)  1.17;12.0(0.21) | 0.88(0.84-0.93)  0.73(0.64-0.81)  0.61(0.51-0.70) |
| Peters 2017[79]  Composite Outcome | Logit  Logit  Logit | 8/35/316  7/35/326  4/35/326 | 0.79;6.37(0.61)  0.82;13.0(0.11)  0.89;6.95(0.54) | 0.83(0.77-0.89)  0.82(0.76-0.88)  0.75(0.66-0.84) | 8/35/316  7/35/326  4/35/326 | | Fair  Fair  Fair | 0.79(0.69-0.88)  0.78(0.67-0.87)  0.73(0.63-0.83) | -  -  - | -  -  - | -  -  - | -  -  - |
| Sub-outcome 1  Incidence CKD | Logit  Logit  Logit | 3/37/300  4/37/300  7/37/276 | 0.93;3.21(0.92)  0.91;4.69(0.85)  0.83;5.70(0.68) | 0.91(0.87-0.94)  0.92(0.88-0.95)  0.92(0.88-0.95) | 3/37/300  4/37/300  7/37/276 | | Fair  Fair  Fair | 0.90(0.83-0.97)  0.90(0.83-0.97)  0.90(0.83-0.97) | -  -  - | -  -  - | -  -  - | -  -  - |
| Sub-outcome 2  eGFR decline ≥30% | Logit  Logit  Logit | 5/30/334  7/30/334  9/9/316 | 0.89;3.03(0.93)  0.84;3.38(0.91)  0.77;7.65(0.47) | 0.84(0.78-0.91)  0.88(0.82-0.93)  0.88(0.82-0.94) | 5/30/334  7/30/334  9/9/316 | | Fair  Fair  Fair | 0.82(0.73-0.91)  0.85(0.76-0.94)  0.84(0.75-0.93) | -  -  - | -  -  - | -  -  - | -  -  - |
| Sub-Outcome 3  eGFR decline ≥5 | Logit  Logit  Logit | 3/28/335  4/28/335  7/28/335 | 0.90;5.82(0.67)  0.86;9.47(0.31)  0.77;8.07(0.43) | 0.76(0.67-0.85)  0.78(0.69-0.87)  0.82(0.74-0.90) | 3/28/335  4/28/335  7/28/317 | | Fair  Fair  Fair | 0.74(0.63-0.85)  0.75(0.64-0.86)  0.78(0.68-0.88) | -  -  - | -  -  - | -  -  - | -  -  - |
| Nowak 2018[23] | Logit | 5/114/ 1032 | Fair | 0.81(0.77-0.85) | 5/114/1032 | | Fair | 0.80(0.77-0.83) | Nowak 2018[23] | 5/ - /462 | - | 0.74(0.65-0.85) |
| Basu 2017[12] | Cox  Cox  Cox  Cox  Cox | 15/627/9635  14/1551/9635  15/5910/9635  14/2321/9635  14/6195/9635 | -  -  -  -  - | -  -  -  -  - | 15/627/9635  14/1551/9635  15/5910/9635  14/2321/9635  14/6195/9635 | | 1.14;79.4(<0.01)  0.94;5.7(0.77)  0.91;42.9(<0.01)  0.96;4.6(0.87)  0.86;74.1(<0.01) | 0.84(0.82-0.86)  0.62(0.61-0.64)  0.76(0.75-0.77)  0.61(0.60-0.63)  0.73(0.72-0.74) | Basu 2017[12] | -  -  -  13/ - /1018  - | -  -  -  1.31;9.3(0.16)  - | -  -  -  0.65(0.61-0.70)  - |
|  |  |  |  |  |  | |  |  | Basu 2018[44] | 13/396/1555  13/280/1746 | 1.04;10.8(0.10)  1.01; 4.2(0.12)  0.99;15.1(0.08)  1.16;9.6(0.05)  0.98;2.9(0.09)  0.97;26.3(<0.01) | 0.85(0.83-0.88)  0.84(0.81-0.87)  0.76(0.73-0.79)  0.71(0.67-0.75)  0.77(0.74-0.80)  0.64(0.60-0.68) |
| Blech 2011[19] | Logit  Logit | 10/556/1274  10/556/1274 | -  - | 0.67(0.64-0.71)  0.56(0.56-0.57) | 10/417/956  10/139/318 | | -  - | 0.67(0.64-0.71)  0.63(0.57-0.69) | Blech 2011[19] | 10/296/906 | -  - | 0.67(0.63-0.71)  0.57(0.54-0.62) |
| Wu 2017[25] | Logit | 4/590/ 4795 | HL 9.4(0.31)  - | 0.71(0.69-0.73) | -  - | | -  - | -  - | Wu 2017[25] | 4/1685/3515  4/462/970 | -  - | 0.72(0.69-0.74)  0.69(0.63-0.76) |
| Dunkler 2015[20] | Logit  Logit | 5/1079/ 6766  15/1079/6766 | Fair; OE=0.98  Fair; OE=0.91 | 0.68(0.66-0.70)  0.69(0.67-0.71) | -  - | | -  - | -  - | Dunkler 2015[20] | 5/1238/8300  15/1238/8300 | Fair; OE=1.01  Fair; OE=1.03 | 0.68(0.66-0.70)  0.69(0.67-0.71) |
| Q-Kidney Score | Logit | - | - | - | - | | - | - | Qian 2019[69] | 6/ - /377 | HL 13.04(0.11) | 0.75(0.69-0.80) |
| 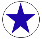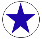 |  |  |  |  |  | |  |  |  |  |  |  |
| Hu 2020[65] | Logit | 8/701/3489 | 0.95(0.93-0.97) | 0.74(0.72-0.76) | 8/701/3489 | | - | 0.73(0.71-0.76) | - | - | - | - |
| Wysham 2020[72] | Logit | 9/6219/89736 | 1.04(0.99-1.09) | 0.71(0.70-0.71) | 9/2564/38458 | | - | 0.70(0.69-0.71) | - | - | - | - |
| Jiang 2020[66] | Logit | 9/100/214 | 1.07(1.04-1.10) | 0.93(0.90-0.96) | 9/42/88 | | - | 0.88(0.80-0.95) | - | - | - | - |
| Liao 2019[67] | Logit  Logit  Logit | 6/246/995  5/246/995  6/246/995 | 1.07(0.85-1.16)  HL 11.9(0.15)  - | 0.78(075-0.81)  0.75(0.72-0.78)  0.64(0.60-0.68) | 6/179/519  5/179/519  6/176/519 | | 0.99(0.76-1.22)  HL 10.5(0.23)  - | 0.70(0.65-0.74)  -  - | -  -  - | -  -  - | -  -  - | -  -  - |
| Romero 2019[70] | RF | 16/4466/6766 | - | 0.81(0.80-0.82) | 16/2311/3485 | | - | 0.88(0.87-0.89) | - | - | - | - |
|  | Logit-1 | 16/4466/6766 | - | 0.77(0.76-0.79) | 16/2311/3485 | | - | 0.86(0.84-0.87) | - | - | - | - |
|  | Logit-2 | 16/4466/6766 | - | 0.80(0.79-0.82) | 16/2311/3485 | | - | 0.89(0.88-0.90) | - | - | - | - |
|  | NB | 16/4466/6766 | - | 0.76(0.75-0.77) | 16/2311/3485 | | - | 0.80(0.78-0.81) | - | - | - | - |
|  | DT | 16/4466/6766 | - | 0.84(0.83-0.85) | 19/2311/3485 | | - | 0.88(0.87-0.89) | - | - | - | - |
| Dagliati 2018[13] | Logit 3y  Logit 5y  Logit 7y | 4/128/ 1000  4/128/ 1000  4/128/ 1000 | -  -  - | 0.70(0.66-0.74)  0.73(0.69-0.78)  0.72(0.67-0.77) | 4/128/ 1000  4/128/ 1000  4/128/ 1000 | | -  -  - | 0.70(0.66-0.74)  0.73(0.69-0.78)  0.72(0.67-0.77) | -  -  - | -  -  - | -  -  - | -  -  - |
|  | RF 3y  RF 5y  RF 7y | 4/128/ 1000  4/128/ 1000  4/128/ 1000 | -  -  - | 0.51(0.49-0.53)  0.51(0.48-0.53)  0.57(0.53-0.62) | 4/128/ 1000  4/128/ 1000  4/128/ 1000 | | -  -  - | 0.51(0.49-0.53)  0.51(0.48-0.53)  0.57(0.53-0.62) | -  -  - | -  -  - | -  -  - | -  -  - |
|  | SVM 3y  SVM 5y  SVM 7y | 4/128/ 1000  4/128/ 1000  4/128/ 1000 | -  -  - | 0.50(0.47-0.54)  0.68(0.65-0.74)  0.71(0.66-0.75) | 4/128/ 1000  4/128/ 1000  4/128/ 1000 | | -  -  - | 0.50(0.47-0.54)  0.68(0.65-0.74)  0.71(0.66-0.75) | -  -  - | -  -  - | -  -  - | -  -  - |
|  | NB 3y  NB 5y  NB 7y | 4/128/ 1000  4/128/ 1000  4/128/ 1000 | -  -  - | 0.50(0.50-0.50)  0.54(0.52-0.56)  0.60(0.56-0.64) | 4/128/ 1000  4/128/ 1000  4/128/ 1000 | | -  -  - | 0.50(0.50-0.50)  0.54(0.52-0.56)  0.60(0.56-0.64) | -  -  - | -  -  - | -  -  - | -  -  - |
| Miao 2017[22] | Cox  Cox | 6/26/3386  8/19/2319 | Fair;10.4(0.11)  Fair;14.3(0.08)  - | 0.84(0.80-0.88)  0.80(0.74-0.86)  0.82(0.82-0.83) | 6/19/3317  8/13/2749 | | Fair;11.2(0.08)  Fair;14.6(0.07)  - | 0.85(0.81-0.90)  0.79(0.74-0.84)  0.81(0.81-0.82) | -  -  - | -  -  - | -  -  - | -  -  - |
| Low 2017[78] | Logit | 6/467/ 1107 | 0.99;0.65(0.98) | 0.80(0.77-0.83) | 6/212/475 | | 0.93;1.36(0.92) | 0.83(0.79-0.87) | - | - | - | - |
| Tanaka 2013[59] | Cox | 5/71/1748 | 1.04; HL (0.11) | 0.76(0.69-0.85) | 5/71/1748 | | - | - | - | - | - | - |
| Jardine 2012[74] | Cox  Cox  Cox  Cox | 8/2715/7286  7/2715/7286  1/2715/7286  1/2715/7286 | Fair; 16.5(0.06)  -  -  - | 0.65(0.64-0.66)  0.63(0.62-0.64)  0.62(0.61-0.63)  0.54(0.53-0.55) | 8/2715/ 7286  -  -  - | | Fair  -  -  - | 0.65(0.63-0.66)  -  -  - | -  -  -  - | -  -  -  - | -  -  -  - | -  -  -  - |
| Goldfarb-R 2002[73] | Logit | 13/40/ 86 | 0.99(0.95-1.04) | 0.81(0.59-1.00) | 13/40/ 86 | | Fair | 0.81(0.59-1.00) | - | - | - | - |
|  | NN | 13/40/ 86 | 1.11(1.04-1.18) | 0.91(0.82-1.00) | 13/40/ 86 | | Fair | 0.91(0.82-1.00) | - | - | - | - |
|  | TB  TB | 13/40/ 86  13/40/ 86 | 0.89(0.87-0.93)  0.88(0.86-0.91) | 0.88(0.64-1.00)  0.87(0.64-1.00) | 13/40/ 86  13/40/ 86 | | Fair  Fair | 0.88(0.64-1.00)  0.87(0.64-1.00) | -  - | -  - | -  - | -  - |
|  | GA | 13/40/ 86 | 1.04(0.99-1.08) | 0.87(0.68-1.00) | 13/40/ 86 | | Fair | 0.87(0.68-1.00) | - | - | - | - |
| 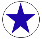 |  |  |  |  |  | |  |  |  |  |  |  |
| Klisic 2018[21] | Logit | 6/40/106 | - | 0.93(0.88-0.98) | - | | - | - | - | - | - | - |
| Jenks 2017[75] | Cox | 8/199/ 701 | - | 0.78(0.72-0.84) | - | | - | - | - | - | - | - |
| Kim 2017[76] | Cox  Cox  Cox | 10/25/325  10/25/325  10/25/325 | -  -  - | 0.80(0.69-0.91)  0.72(0.60-0.84)  0.77(0.66-0.88) | -  -  - | | -  -  - | -  -  - | -  -  - | -  -  - | -  -  - | -  -  - |
| Lindhart 2017[77] | Cox  Cox  Cox | 10/89/737  5/89/737  4/89/737 | -  -  - | 0.79(0.75-0.84)  0.79(0.74-0.83)  0.76(0.70-0.81) | -  -  - | | -  -  - | -  -  - | -  -  - | -  -  - | -  -  - | -  -  - |
| Saulnier 2017[81]  Renal Function Loss | Cox  Cox  Cox  Cox  Cox  Cox  Cox | 10/397/1135  8/397/1135  8/397/1135  8/397/1135  9/397/1135  9/397/1135  9/397/1135 | -  -  -  -  -  -  - | 0.77(0.74-0.80)  0.76(0.73-0.79)  0.75(0.72-0.78)  0.74(0.71-0.78)  0.77(0.74-0.80)  0.76(0.73-0.79)  0.76(0.73-0.79) | -  -  -  -  -  -  - | | -  -  -  -  -  -  - | -  -  -  -  -  -  - | -  -  -  -  -  -  - | -  -  -  -  -  -  - | -  -  -  -  -  -  - | -  -  -  -  -  -  - |
| Rapid Renal Decline | Cox  Cox  Cox  Cox  Cox  Cox  Cox | 10/233/1109  8/233/1109  8/233/1109  8/233/1109  9/233/1109  9/233/1109  9/233/1109 | -  -  -  -  -  -  - | 0.79(0.76-0.83)  0.78(0.75-0.82)  0.78(0.75-0.82)  0.75(0.71-0.79)  0.79(0.75-0.82)  0.80(0.76-0.83)  0.79(0.75-0.82) | -  -  -  -  -  -  - | | -  -  -  -  -  -  - | -  -  -  -  -  -  - | -  -  -  -  -  -  - | -  -  -  -  -  -  - | -  -  -  -  -  -  - | -  -  -  -  -  -  - |
| Yang 2017[82] | Logit  Logit  Logit | 9/110/210  8/110/210  8/110/210 | -  -  - | 0.78(0.68-0.88)  0.76(0.64-0.88)  0.76(0.62-0.84) | -  -  - | | -  -  - | -  -  - | -  -  - | -  -  - | -  -  - | -  -  - |
| Zobel 2017[83] | Cox  Cox | 9/42/200  8/42/200 | -  - | 0.75(0.66-0.84)  0.72(0.63-0.81) | -  - | | -  - | -  - | -  - | -  - | -  - | -  - |
| Riphagen 2015[80] | Cox  Cox | 8/183/640  8/79/1143 | Fair  Fair | 0.69(0.65-0.72)  0.73(0.68-0.78) | -  - | | -  - | -  - | -  - | -  - | -  - | -  - |
| Welsh 2014[24] | Cox  Cox  Cox  Cox | 18/283/3225  17/283/3225  17/283/3225  17/283/3225 | -  -  -  - | 0.84(0.80-0.88)  0.83(0.80-0.87)  0.82(0.79-0.86)  0.84(0.80-0.87) | -  -  -  - | | -  -  -  - | -  -  -  - | -  -  -  - | -  -  -  - | -  -  -  - | -  -  -  - |
| **End Stage Renal Disease (n = 18)** | | | | | | | | | | | | |
|  |  | **17/51/0** | | | **7/11/0** | | | |  | **4/13/0** | | |
| 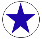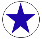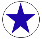 | | | | | | | | | | | | |
| Basu 2017[12] | Cox | 14/292/9635 | - | - | 14/292/ 9635 | | 1.28;30.8(<0.01) | 0.60(0.56-0.64) | Basu 2017[12]  UKPDS-OM2 | 14/184/1018  14/184/1018 | -  - | -  0.54(0.50-0.59) |
|  |  |  |  |  |  | |  |  | Basu 2018[44] | 14/396/1555  14/396/1555  14/280/1746  14/280/1746 | -  0.83;13.9(0.05)  -  1.02;42.1(<0.01) | 0.78(0.75-0.81)  0.77(0.74-0.80)  0.91(0.89-0.93)  0.68(0.64-0.72) |
| Wan 2017[29] | Cox  Cox | 16/182/36289  17/141/41347 | - | - | 16/91/18145  17/71/20674 | | -  - | 0.86(0.85-0.88)  0.86(0.84-0.88) | Wan 2017[29]  (Advanced -New Zealand Equation) | 11/91/18145  12/70/20674  11/91/18145  12/70/20674 | - | 0.85(0.84-0.88)  0.84(0.82-0.87)  0.86(0.85-0.88)  0.86(0.84-0.88) |
| Elley 2013[26] | Cox (4)  Cox (3)  Cox (2)  Cox (1) | 12/637/25736  8/637/25736  10/637/25736  6/637/25736 | -  -  -  - | 0.89(0.87-0.91)  0.88(0.87-0.90)  0.89(0.87-0.91)  0.88(0.87-0.90) | -  -  -  - | | -  -  -  - | -  -  -  - | Elley 2013[26] | 12/121/5877  8/121/5877  10/121/5877  6/121/5877 | HL (p=0.66)  HL (p=0.69)  HL (p=0.69)  HL (p=0.32) | 0.91(0.88-0.94)  0.92(0.88-0.93)  0.92(0.89-0.94)  0.86(0.82-0.89) |
| 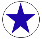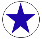 |  |  |  |  |  | |  |  |  |  |  |  |
| Wysham 2020[72] | Logit  Logit | 6/1619/112023  10/2135/112023 | 1.02(0.99-1.04)  1.05(1.03-1.07) | 0.82(0.80-0.83)  0.85(0.84-0.86) | 6/672/48008  10/912/48008 | | - | 0.82(0.80-0.84)  0.84(0.82-0.86) | -  - | -  - | -  - | -  - |
| Lin 2017[84] | Cox | 11/1215/ 24104 | Fair;HL (p>0.05) | 0.91(0.88-0.92) | 11/1215/24104 | | Fair; HL (p>0.05) | 0.92(0.90-0.93) | - | - | - | - |
| Li 2016[28] | Cox  Cox | 4/22/604  4/22/604 | -  - | 0.94(0.89-0.99)  0.90(0.82-0.98) | 4/22/604  4/22/604 | | -  - | 0.88(0.78-0.97)  - | -  - | -  - | -  - | -  - |
| Jardine 2012[74] | Cox | 7/166/ 10506 | - | 0.85(0.82-0.88) | 7/166/10506 | | 0.99(0.981.02) | 0.86(0.82-0.89) | - | - | - | - |
| Yang 2006[85] | Cox  Cox  Cox | 8/72/2227  7/72/2227  4/72/2227 | -  -  - | 0.97(0.95-1.00)  0.96(0.94-1.00)  0.88(0.83-0.93) | 8/87/ 2211  7/87/ 2211  4/87/ 2211 | | Fair; HL(p>0.05)  -  - | 0.96(0.94-0.99)  0.96(0.93-0.98)  0.84(0.80-0.88) | -  -  - | -  -  - | -  -  - | -  -  - |
| 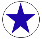 |  |  |  |  |  | |  |  |  |  |  |  |
| Garlo 2018[27] | Cox  Cox  Cox  Cox  Cox  Cox | 10/98/5367  10/98/5367  10/98/5367  9/98/5367  9/98/5367  10/98/5367 | Fair;3.87(>0.05)  Fair;5.14(>0.05)  Fair;3.55(>0.05)  Fair;5.13(>0.05)  Fair;5.31(>0.05)  Fair;3.76(>0.05) | 0.97 (0.94-0.99)  0.93 (0.86-0.96)  0.93 (0.86-0.96)  0.93 (0.86-0.96)  0.94 (0.90-0.97)  0.97 (0.94-0.99) | -  -  -  -  -  - | | -  -  -  -  -  - | -  -  -  -  -  - | -  -  -  -  -  - | -  -  -  -  -  - | -  -  -  -  -  - | -  -  -  -  -  - |
| Mise 2017[90] | Cox  Cox  Cox | 9/104/136  8/104/136  8/104/136 | -  -  - | 0.78(0.74-0.82)  0.77(0.73-0.82)  0.76(0.71-0.80) | -  -  - | | -  -  - | -  -  - | -  -  - | -  -  - | -  -  - | -  -  - |
| Mise 2016[89] | Cox  Cox | 8/94/149  7/94/149 | -  - | 0.84(0.80-0.88)  0.82(0.78-0.86) | -  - | | -  - | -  - | -  - | -  - | -  - | -  - |
| Foster 2015[86] | Cox  Cox  Cox  Cox | 10/69/250  10/69/250  10/69/250  10/69/250 | -  -  -  - | 0.85(0.84-0.85)  0.84(0.83-0.85)  0.84(0.83-0.85)  0.84(0.83-0.85) | -  -  -  - | | -  -  -  - | -  -  -  - | -  -  -  - | -  -  -  - | -  -  -  - | -  -  -  - |
| Fufaa 2014[87] | Cox  Cox  Cox  Cox | 9/74/260  9/74/260  9/74/260  9/74/260 | -  -  -  - | 0.84(0.82-0.85)  0.82(0.82-0.84)  0.83(0.82-0.87)  0.83(0.82-0.86) | -  -  -  - | | -  -  -  - | -  -  -  - | -  -  -  - | -  -  -  - | -  -  -  - | -  -  -  - |
| Pavkov 2013[92] | Cox  Cox  Cox | 8/68/234  8/68/234  8/68/234 | -  -  - | 0.85(0.82-0.87)  0.81(0.78-0.84)  0.81(0.78-0.84) | - -  - | | -  -  - | -  -  - | -  -  - | -  -  - | -  -  - | -  -  - |
| Niewczas 2012[91] | Cox  Cox | 6/59/410  6/59/410 | -  - | 0.93(0.89-0.97)  0.84(0.78-0.90) | -  - | | -  - | -  - | -  - | -  - | -  - | -  - |
| Heersprink 2010[88] | Cox  Cox  Cox  Cox | 4/202/701  4/202/701  4/202/701  4/202/701 | -  -  -  - | 0.82(0.79-0.86)  0.78(0.74-0.82)  0.78(0.75-0.82)  0.79(0.75-0.83) | -  -  -  - | | -  -  -  - | -  -  -  - | -  -  -  - | -  -  -  - | -  -  -  - | -  -  -  - |
| Keane 2006[93] | Cox  Cox | 4/341/1513  5/555/1513 | -  - | -  - | -  - | | -  - | -  - | -  - | -  - | -  - | -  - |

**Abbreviations:** AB; Adaptive Boosting; BS, Backward Stepwise; BN, Bayesian Network; DT, Decision Tree; GA, Generalised additive; GBM, Gradient Boosting Machine; GLM; Generalized Linear Model; HL; Hosmer-Lemeshow Chi Square test; k-NN, k-nearest neighbour; LR, Logit regression; NB, Naïve Bayesian; NN, Neural Network; O/E, Observed per expected; RB, RUS Boost; RF, Random Forest; SVM, Support Vector Machine

**Notes:**

Calibration: O/E ratio (95%CI) or Calibration slope, Hosmer-Lemeshow χ^2^ (p-value), or Calibration plot (fairness)

N_1_: Number of studies; N2, Number of traditional statistical models; N3, number of Machine learning models


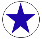

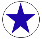

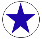
 Evidences of derive-all validations/derive-external validations,
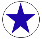

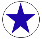
 Evidences of derive-internal validations,
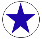
 Evidences of Derivative only

### **TABLE S3** Risk of bias assessment using PROBAST

| **Study, Year** | **Participant selection** | **Predictors** | **Outcome** | **Sample Size & Participants flow** | **Analysis** | **Overall**  **Risk-of bias** |
| --- | --- | --- | --- | --- | --- | --- |
| **Diabetic Retinopathy** | | | | | | |
| Yusufi 2019[43] | 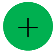 | 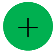 | 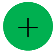 | 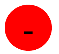 | 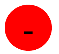 | 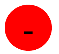 |
| Liao 2018 [42] | 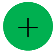 | 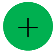 | 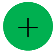 | 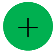 | 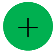 | 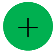 |
| Basu 2018[44] | 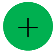 | 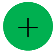 | 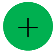 | 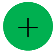 | 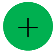 | 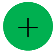 |
| Basu 2017[12] | 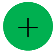 | 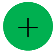 | 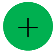 | 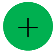 | 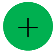 | 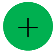 |
| Cox 2015[41] | 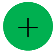 | 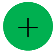 | 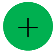 | 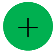 | 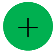 | 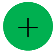 |
| Pedre 2015[46] | 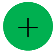 | 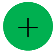 | 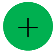 | 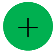 | 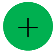 | 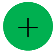 |
| Heijden 2014[30] | 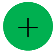 | 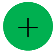 | 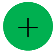 | 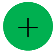 |  |  |
| Oh E 2013[17] |  |  |  |  |  |  |
| Wang 2014[47] |  |  |  |  |  |  |
| Aspelund 2011[11] |  |  |  |  |  |  |
| Hosseini 2009[45] |  |  |  |  |  |  |
| Mo 2020[49] |  |  |  |  |  |  |
| Zhu 2020[53] |  |  |  |  |  |  |
| Ochs 2019[50] |  |  |  |  |  |  |
| Ougunyemi 2019[51] |  |  |  |  |  |  |
| Tsao 2018[52] |  |  |  |  |  |  |
| Dagliati 2018[13] |  |  |  |  |  |  |
| Finana 2018[48] |  |  |  |  |  |  |
| Herrera 2017[57] |  |  |  |  |  |  |
| Soleiman 2015[54] |  |  |  |  |  |  |
| Ogunyemi 2015[15] |  |  |  |  |  |  |
| Ogunyemi 2013[16] |  |  |  |  |  |  |
| Tanaka 2013[59] |  |  |  |  |  |  |
| Semeraro 2011[18] |  |  |  |  |  |  |
| Ng 2008[58] |  |  |  |  |  |  |
| Han 2004[56] |  |  |  |  |  |  |
| Chong 2017[55] |  |  |  |  |  |  |
| Mogilevskyy 2017[62] |  |  |  |  |  |  |
| Kengne 2015[14] |  |  |  |  |  |  |
| Cichosz 2015[61] |  |  |  |  |  |  |
| Welsh 2014[24] |  |  |  |  |  |  |
| Bresnick 1987[60] |  |  |  |  |  |  |
| **Diabetic Nephropathy**  **Sub Outcome Chronick Kidney Disease** | | | | | | |
| Song 2019[71] |  |  |  |  |  |  |
| Peters 2019[68] |  |  |  |  |  |  |
| Qian 2019[69] |  |  |  |  |  |  |
| Nowak 2018[23] |  |  |  |  |  |  |
| Blech 2011[19] |  |  |  |  |  |  |
| Miao 2017[22] |  |  |  |  |  |  |
| Wu 2017[25] |  |  |  |  |  |  |
| Dunkler 2015[20] |  |  |  |  |  |  |
| Hu 2020[65] |  |  |  |  |  |  |
| Wysham 2020[72] |  |  |  |  |  |  |
| Jiang 2020[66] |  |  |  |  |  |  |
| Liao 2019[67] |  |  |  |  |  |  |
| Romero 2019[70] |  |  |  |  |  |  |
| Low 2017[78] |  |  |  |  |  |  |
| Peters 2017[79] |  |  |  |  |  |  |
| Jardine 2012[74] |  |  |  |  |  |  |
| Goldfarb 2002[73] |  |  |  |  |  |  |
| Klisic 2018[21] |  |  |  |  |  |  |
| Jenks 2017[75] |  |  |  |  |  |  |
| Kim 2017[76] |  |  |  |  |  |  |
| Lindhart 2017[77] |  |  |  |  |  |  |
| Saulnier 2017[81] |  |  |  |  |  |  |
| Yang 2017[82] |  |  |  |  |  |  |
| Zobel 2017[83] |  |  |  |  |  |  |
| Riphagen 2015[80] |  |  |  |  |  |  |
| **Sub Outcome End Stage Renal Disease (ESRD)** | | | | | | |
| Wan 2017[29] |  |  |  |  |  |  |
| Elley 2013[26] |  |  |  |  |  |  |
| Lin 2017[84] |  |  |  |  |  |  |
| Li 2016[28] |  |  |  |  |  |  |
| Yang 2006[85] |  |  |  |  |  |  |
| Garlo 2018[27] |  |  |  |  |  |  |
| Mise 2017[90] |  |  |  |  |  |  |
| Mise 2016[89] |  |  |  |  |  |  |
| Foster 2015[86] |  |  |  |  |  |  |
| Fufaa 2015[87] |  |  |  |  |  |  |
| Pavkov 2013[92] |  |  |  |  |  |  |
| Niewczas 2012[91] |  |  |  |  |  |  |
| Heersprink 2010[88] |  |  |  |  |  |  |
| Keane 2006[93] |  |  |  |  |  |  |

: Unclear Risk : High Risk : Low Risk

### **TABLE S4** Prognostic equations that were externally validated

| **Study, Year** | **Prognostic Models** | **Equations** | **External Validation** | |
| --- | --- | --- | --- | --- |
|  |  |  | **Study, Year** | **C-statistic (95%CI)** |
| **Diabetic Retinopathy (DR)** | | | | |
| Liao 2018[42] | Multi-Locus Genetic Risk Score | $\begin{matrix} \text{GRS = 4/3.462 }\times\text{ [(ARHGAP22\_rs11101385\_Risk }\times\text{ 0.932)] } \\ \text{ + (FMN1\_rs142644390\_Risk }\times\text{ 1.077) } \\ \text{ + (PLXDC2\_rs4748644\_Risk }\times\text{ 0.451) } \\ \text{ + (CNTN5\_rs61893374\_Risk }\times\text{ 1.002)]} \end{matrix}$ $\begin{matrix} \text{Score = Age + (3.49 × DD}_{>10}\text{) + (1.90 × HbA1c}_{>8}\text{) + (2.04 × SBP}_{>140}\text{) } \\ \text{ + (1.30 × GRS}_{Q2(3.01-3.53)}\text{) + (2.75 × GRS}_{Q3(>3.54)}\text{) } \end{matrix}$ | Liao 2018[42] | 0.66(0.60-0.72) ^⸷^ |
| Basu 2017[12] | RECODE | $\begin{matrix} \text{Score = (0.009 }\times\text{ Age) + (0.225 }\times\text{ Female) - (0.065 }\times\text{ Black) + (0.010 }\times\text{ SBP) } \\ \text{ + (0.218 }\times\text{ CVD) + (0.180 }\times\text{ BP drug) - (0.317 }\times\text{ Oral DM drugs) } \\ \text{ + (0.172 }\times\text{ HbA1c) - (0.001 }\times\text{ TC) + (0.006 }\times\text{ HDL) + (0.844 }\times\text{ S-Cr) } \\ \text{DR = (1 - 0.973}^{\text{Score}}\text{) }\times\text{ 100\%} \end{matrix}$ | Basu 2017[12]  Basu 2018[44] | 0.59 (0.57-0.62) ^‡^  0.76 (-) ^‡^  0.64 (-) ^‡^ |
| Hosseini 2019[45] | Iranian DR Risk Score | $\begin{matrix} \text{Score = (5 × HbA1c}_{\text{(7-9)}}\text{) + (5 × HbA1c}_{\text{(9-11)}}\text{) + (10 × HbA1c}_{\text{(>11)}}\text{) + (15 × Age}_{\text{(40-49)}}\text{) } \\ \text{ + (25 × Age}_{\text{(50-59)}}\text{) + (25 × Age}_{\text{(³60)}}\text{) + (20 }\times\text{ DD}_{(2-4)}\text{) + (35 }\times\text{ DD}_{(5-9)}\text{) } \\ \text{ + (50 }\times\text{ DD}_{(\geq10)}\text{) + (10 }\times\text{ BMI}_{(>25)}\text{) + (5 }\times\text{ Female)} \end{matrix}$ | Yusufi 2019 | 0.82 (0.76-0.86) ^‡^ |
| Wang 2014[47] | Chinese DR Risk Score | $\begin{matrix} \text{Score = (5 × Age}_{\text{(45-64)}}\text{) + (4 × Age}_{\text{(}\geq\text{65)}}\text{) + (2 × Central Obesity) + (9 }\times\text{ DD}_{(1-5)}\text{) } \\ \text{ + (10 }\times\text{ DD}_{(5-10)}\text{) + (14 }\times\text{ DD}_{(10-15)}\text{) + (20 }\times\text{ DD}_{(>15)}\text{) } \\ \text{ + (7 }\times\text{ Anti Hypertensive drug)} \end{matrix}$ | Yusufi 2019 | 0.78 (0.73-0.83) ^‡^ |
| Oh 2013[17] | - | $\begin{matrix} \text{DR = 2.047 - (0.012 }\times\text{ Age) + (0.469 }\times\text{ Current smoke) - (0.137 }\times\text{ Alcohol) } \\ \text{ - (0.082 }\times\text{ BMI) + (0.027}\times\text{ DD) + (0.427 }\times\text{ Diagnosed DM) } \\ \text{ + (0.956 }\times\text{ Insulin) - (0.028 }\times\text{ HLD) - (0.004 }\times\text{DBP)} \\ \text{ + (0.008 }\times\text{ FPG) + (0.054}\times\text{ HbA1c) + (0.256 }\times\text{ Hb) } \\ \text{ + (0.002 }\times\text{TG) + (0.037 }\times\text{ BUN) + (0.141 }\times\text{ PRO) } \\ \text{ + (0.442 }\times\text{ GLU) + (0.012 }\times\text{ Bilirubin) + (0.096 }\times\text{Blood) } \\ \text{ - (0.111 }\times K\text{eton)} \end{matrix}$ | Oh 2013[17] | 0.82(0.78-0.85) ^⸷^ |
| Aspelund 2011[11] | STR risk score |  | Pedre 2015[46]  Heijden 2014[30] | 0.74(0.62-0.85) ^‡^  0.83(0.74-0.92) ^‡^ |
| **Chronic Kidney Disease (CKD)** | | | | |
| Nowak 2018[23] | Early renal decline risk score | $\text{CKD = ACR}^{0.26}\text{ }\times\text{ SBP}^{2.35}\text{ }\times\text{ TNFR1}^{1.05}\text{ }\times\text{ (KIM1 }\times\text{ (MCP1/EGF))}^{0.42}\text{ }\times\text{ 10}^{\text{-3}}$ | Nowak 2018[23] | 0.74 (0.65-0.85)^⸷ ⸹^ |
| Basu 2017[12] | RECODE^‡^ | $\begin{matrix} \text{Score = (0.019}\times\text{Age) + (0.103}\times\text{Female) - (0.026}\times\text{Black) + (0.241}\times\text{Hisp/Latin)} \\ \text{ + (0.265 }\times\text{Current Smoke) + (0.001 }\times\text{SBP) + (0.181 }\times\text{ CVD) } \\ \text{ + (0.294 }\times\text{ BP drug) + (0.047 }\times\text{Oral DM drugs) } \\ \text{ + (0.422 }\times\text{Anticoagulants) + (0.133 }\times\text{HbA1c) + (0.0003 }\times\text{ TC) } \\ \text{ - (0.006 }\times\text{ HDL) + (0.461 }\times\text{ S-Cr)} \\ \text{CKD = (1 - 0.973}^{\text{Score}}\text{) }\times\text{ 100\%} \end{matrix}$ | Basu 2017[12]  Basu 2018[44] | 0.65 (0.61-0.70) ^‡^  0.76 (-) ^‡^  0.71 (-) ^‡^ |
| Wu 2017[25] | Diabetic Kidney Disease (DKD) Risk Score | $\begin{matrix} \text{CKD = (0.525 }\times\text{ Male) + (0.322}\times\text{ BMI}_{\text{(<25)}}\text{) + (0.602}\times\text{ BMI}_{\text{(}\geq\text{28)}}\text{) + (0.626}\times\text{ SBP}_{\text{(120-129)}}) \\ \text{ + (0.970}\times\text{ SBP}_{\text{(130-139)}}\text{)+ (1.732}\times\text{ SBP}_{\text{(}\geq\text{140)}}\text{) + (0.322}\times\text{ DD}_{\text{(5-9.9) }}\text{) + (0.794}\times\text{ DD}_{\text{(10-14.9)}}) \\ \text{ + (1.074}\times\text{ DD}_{\text{(>15)}}) \end{matrix}$ | Wu 2017[25] | 0.72 (0.69-0.74)^⸷^  0.69 (0.63-0.76)^⸷^ |
| Dunkler 2015[20] | Laboratory Models | $\begin{matrix} \text{CKD = -0.76727 - (0.181 }\times\text{ Albuminuria) - 5.449 }\times\text{ }\left[\left(\text{d-UACR}_{\text{tp}}\text{+0.1}\right)\right]\text{+4.726 } \\ \text{ }\times\left[\left(\text{d-UACR}_{\text{tp}}\text{+0.1}\right)\text{/5}\right]^{3}\text{+ 0.089}\left[\left(\text{eGFR+0.1}\right)\text{/120}\right]^{\text{-2}}\text{+ 0.008 }\times\text{ Female } \\ \text{ - 0.208}\left[\left(\text{Age+0.1}\right)\text{/90}\right] \end{matrix}$ | Dunkler 2015[20] | 0.66 (0.63-0.69) ^‡^ |
|  | Clinical Models | $\begin{matrix} \text{CKD = -0.7382 - 0.221}\times\text{ Albuminuria - 4.831}\times\text{ }\left[\left(\text{d-UACR}_{\text{tp}}\text{+0.1}\right)\text{/5}\right]\text{+4.469} \\ \text{ }\times\left[\left(\text{d-UACR}_{\text{tp}}\text{+0.1}\right)\text{/5}\right]^{3}\text{+ 0.077}\times\text{ }\left[\left(\text{eGFR+0.1}\right)\text{/120}\right]^{\text{-2}}\text{+0.752}\times\text{ }\left[\left(\text{Age+0.1}\right)\text{/90}\right]\text{ } \\ \text{ + 0.362x PAD - 1.145}\times\text{ }\left[\left(\text{Glucose+0.1}\right)\text{/20}\right]^{\text{-1}}\text{-0.504}\times\text{ }\left[\left(\text{Glucose+0.1}\right)\text{/20}\right]^{\text{-1}} \\ \text{ ln}\left[\left(\text{Glucose+0.1}\right)\text{/20}\right]\text{+0.766}\times\text{nDrugs/5 +0.309}\times\text{Asian+0.221}\times\text{Other+0.006} \\ \text{ }\times\left[\text{(LDL+0.1)/10}\right]^{\text{-2}}\times1.319\left[\left(\text{LDL+0.1}\right)\text{/10}\right]^{2}\text{+0.327}\times\left[\text{ln}\left(\text{DD+0.003}\right)\text{+8}\right]\text{/12 } \\ \text{ +0.086}\times\text{ Stroke -0.021}\times\text{ Female+ 0.108 }\times\left[\left(\text{Waist+0.1}\right)\text{/140}\right]^{\text{-2}}\text{+ 0.922} \\ \text{ }\times\left[\left(\text{Waist+0.1}\right)\text{/140}\right]^{\text{-3}}\text{-0.105 MACE -0.101}\times\text{ Laser therapy DR} \end{matrix}$ | Dunkler 2015[20] | 0.68(0.65-0.70) ^‡^ |
| **End stage renal disease (ESRD)** | | | | |
| Basu 2017[12] | RECODE^‡^ | $\begin{matrix} \text{Score = (0.019}\times\text{Age) + (0.011}\times\text{Female) + (0.088}\times\text{Black) +(0.233}\times\text{Hisp/Latin)} \\ \text{ + (0.148}\times\text{Current smoke) + (0.003}\times\text{SBP) +(0.021}\times\text{CVD) + (0.079}\times\text{BP drug) } \\ \text{ + (0.125}\times\text{Oral DM drugs) + (0.031}\times\text{ Anticoagulants)+ (0.136}\times\text{HbA1c) } \\ \text{ + (0.001}\times\text{TC) + (0.006}\times\text{HDL) + (0.861}\times\text{S-Cr) + (0.0003}\times\text{UACR)} \\ \text{ESRD = (1 - 0.99558399}^{\text{exp(Score)}}\text{)x 100\%} \end{matrix}$ | Basu 2017[12]  Basu 2018[44] | 0.60(0.56-0.64) ^‡^  0.78 (-) ^‡^  0.91 (-) ^‡^ |
| Wan 2017[29] |  | $\begin{matrix} \text{Score = (0.058}\times\text{ Age) + (0.254}\times\text{Smoke) + (0.385}\times DR)\text{+ (0.43}\times\text{HT drug)} \\ \text{ + (0.314}\times\text{ Oral DM drug) + (2.28}\times\text{ Insulin) - (0.235}\times\text{ HbA1c) } \\ \text{ + (0.009}\times\text{ SBP) - (0.072}\times\text{ DBP) + (1.147}\times\text{ ln(UACr))} \\ \text{ + (0.896}\times\text{ eGFR}_{\text{(60-89)}}\text{)+ (2.17}\times\text{eGFR}_{\text{(<60)}}\text{) } \\ \text{ - (0.03}\times\text{ (Age }\times\text{ Insulin) - (0.01}\times\text{ (Age }\times\text{ Insulin))} \\ \text{ESRD}_{\text{(M)}}\text{= (1 – 0.9859326}^{\exp(Score)})\times100\% \end{matrix}$ | Wan 2017[29] | 0.87(0.85-0.88)^⸷^ |
|  |  |  |  | 0.86(0.84-0.88)^⸷^ |
| Elley 2013[26] | Diabetic Cohort Study Renal Risk Score | $\begin{matrix} \text{Score = (0.401}\times\text{Female) + (0.821}\times\text{Maori) + (0.296}\times\text{Pacific) - (0.138}\times\text{EastAsian)} \\ \text{ - (0.004}\times\text{IndoAsian) - (0.223}\times\text{Other Ethnicity) + (0.024}\times\text{Age onset) } \\ \text{ + (0.043}\times\text{ DD) + (0.305}\times\text{ S-Cr/10) + (0.644}\times\text{ Microalbuminuria) } \\ \text{ + (1.303}\times\text{Macroalbuminuria) + (2.291}\times\text{ Albuminuria) + (0.049}\times\text{SBP/10)} \\ \text{ + (0.173}\times\text{ HbA1c)}\begin{matrix} \text{- (0.003} \end{matrix}\times\text{ Ex-Smoke)+ (0.252}\times\text{Current Smoke) } \\ \text{ + (0.498}\times\text{ Previous CVD)} \\ \text{ESRD= (1- 0.99558399}^{\text{exp(Score)}}\text{)}\times\text{ 100\%} \end{matrix}$ | Elley 2013[26] | 0.91(0.88-0.94)^⸷^ |

**Abbreviations:** ACR, Albumin creatinine ratio; BMI, body mass index; CKD, chronic kidney disease; CVD, cardiovascular diseases; DBP, diastolic blood pressure; DD, diabetic duration; DLP, dyslipidemia; DR, diabetic retinopathy; EGF, epidermal growth factor (ng/ml);eGFR, estimated glomerular filtration rate (ml/min/1.73 m^2^); F, Female; FPG, fasting plasma glucose; GLU, Glucose; HbA1c, glycated haemoglobin (%); HDL, high density lipoprotein; HDL-C, high density lipoprotein cholesterol; HLD, hyperlipidemia; HT, hypertension; KIM-1, kidney injury molecule-1 (ng/ml);LDL, low density lipoprotein; M, Male; MCP-1, monocyte chemoattractant protein-1 (ng/ml); PA, physical activity; PRO, Protein; RECODE; Risk Equations for Complications Of type 2 Diabetes; SBP, systolic blood pressure; S-Cr, serum creatinine; STR, Sight Threatening Retinopathy; T1D, type 1 diabetes mellitus; TC, total cholesterol; TG, triglycerides, TNFR-1, tumour necrosis factor receptor 1 (ng/ml);UACR, urine albumin creatinine ratio

Notes:^⸷^) externally validated by narrow population ^‡^) validated by other cohort prospective/ retrospective studies ^⸹^ externally validated in T1D population

### **TABLE S5** Summary of pooled C-statistic of prognostic model

| **Study design** | **Statistical Method** | **Phase of Prognostic Model** | | | | | |
| --- | --- | --- | --- | --- | --- | --- | --- |
|  |  | **Derivative** | | **Internal Validation** | | **External Validation** | |
|  |  | **Study** | **C-statistics (95%CI)** | **Study** | **C-statistics (95%CI)** | **Study** | **C-statistics (95%CI)** |
| **Diabetic retinopathy (DR)** | | | | | | | |
| Cohort | Logit | Ogunyemi 2019[51]  Dagliati 2018[13]  Herrera 2017[57]  Cichosz 2015[61]  Ng 2008[58]  Han 2004[56] | 0.758(0.750-0.766)  0.808(0.772-0.845)  0.780(0.747-0.813)  0.740(0.651-0.829)  0.950(0.940-0.960)  0.900(0.883-0.917) | Ogunyemi 2019[51]  Dagliati 2018[13]  Herrera 2017[57]  Ng 2008[58]  Han 2004[56] | 0.752(0.741-0.763)  0.808(0.772-0.845)  0.778(0.673-0.883)  0.920(0.878-0.962)  0.875(0.827-0.923) | - | - |
|  |  | Pooled: I^2^ = 99.47 | 0.825(0.734-0.916) | Pooled: I^2^ = 95.11 | 0.828(0.755-0.901) | - | - |
|  | Cox | Welsh 2014[24]  Aspelund 2011[11]  Semeraro 2011[18] | 0.731(0.689-0.773)  0.760(0.740-0.780)  0.746(0.716-0.776) | - | - | - | - |
|  |  | Pooled: I^2^ = 0 | 0.753(0.737-0.768) |  |  |  |  |
| Cross-sectional | Logit | Mo 2020[49]  Zhu 2020[53]  Tsao 2018[52]  Kengne 2015[14]  Wang 2014[47]  Oh 2013[17]  Hosseini 2009[45] | 0.700(0.676-0.724)  0.884(0.831-0.937)  0.756(0.699-0.813)  0.881(0.828-0.933)  0.700(0.671-0.729)  0.820(0.750-0.890)  0.704(0.685-0.723) | Mo 2020[49]  Zhu 2020[53]  Tsao 2018[52]  Soleiman 2015[54]  Wang 2014[47]  Oh 2013[17] | 0.715(0.674-0.756)  0.803(0.674-0.932)  0.802(0.683-0.921)  0.760(0.710-0.810)  0.626(0.577-0.675)  0.810(0.749-0.869) | Yusufi 2019[43] - Wang  Yusufi 2019[43] - Hosseini  Oh 2013[17] | 0.776(0.734-0.832)  0.815(0.765-0.859)  0.820(0.780-0.850) |
|  |  | Pooled: I^2^ = 93.21 | 0.773(0.722-0.824) | Pooled: I^2^ = 82.46 | 0.744(0.683-0.805) | Pooled: I^2^ = 8.05 | 0.807(0.783-0.832) |
| Genetic risk score | Logit | Liao 2018[42]  Chong 2017[55]  Mogilevskyy 2017[62] | 0.770(0.729-0.811)  0.740(0.710-0.760)  0.700(0.620-0.760) | - | - | - | - |
|  |  | Pooled: I^2^ = 36.73 | 0.744(0.714-0.773) |  |  |  |  |
| **Chronic Kidney Disease (CKD)** | | | | | | | |
| Cohort | Logit | Hu 2020[65]  Wysham 2020[72]  Jiang 2020[66]  Peters 2019[68]  Nowak 2018[23]  Dagliati 2018[13]  Peters 2017[79]  Yang 2017[82]  Wu 2017 [25]  Low 2017[78]  Dunkler 2015[20] | 0.744(0.724-0.764)  0.710(0.703-0.717)  0.934(0.904-0.964)  0.890(0.850-0.940)  0.810(0.770-0.850)  0.734(0.689-0.779)  0.830(0.770-0.890)  0.785(0.683-0.888)  0.713(0.692-0.743)  0.800(0.770-0.830)  0.660(0.624-0.696) | Hu 2020[65]  Wysham 2020[72]  Jiang 2020[66]  Peters 2019[68]  Nowak 2018[23]  Dagliati 2018  Peters 2017[79]  Low 2017[78] | 0.737(0.714-0.760)  0.700(0.689-0.711)  0.875(0.800-0.950)  0.870(0.795-0.945)  0.800(0.785-0.815)  0.734(0.689-0.779)  0.790(0.698-0.882)  0.830(0.790-0.870) | Peters 2019[68]  Nowak 2018[23]  Wu 2017[25]  Dunkler 2015[20] | 0.880(0.840-0.930)  0.740(0.650-0.850)  0.720(0.696-0.744)  0.680(0.653-0.707) |
|  |  | Pooled: I^2^ = 96.91 | 0.782(0.736-0.827) | Pooled: I^2^ = 95.53 | 0.786(0.740-0.832) | Pooled: I^2^ = 94.68 | 0.754(0.674-0.835) |
|  | Cox | Zobel 2017[83]  Saulnier 2017[81]  Miao 2017[22]  Jenks 2017[75]  Riphagen 2015[80]  Welsh 2014[24]  Kim 2017 | 0.752(0.664-0.840)  0.769(0.739-0.799)  0.823(0.820-0.826)  0.783(0.728-0.837)  0.690(0.650-0.720)  0.842(0.809-0.874)  0.800(0.694-0.906) | - | - | - | - |
|  |  | Pooled: I^2^ = 91.78 | 0.781(0.738-0.823) | - | - | - | - |
| RCT | Cox | Lindhart 2017[77]  Tanaka 2013[59]  Jardine 2012[74] | 0.790(0.750-0.840)  0.767(0.690-0.845)  0.647(0.637-0.658) | - | - | - | - |
|  |  | Pooled: I^2^ = 95.53 | 0.732(0.621-0.843) | - | - | - | - |
| **End Stage Renal Disease (ESRD)** | | | | | | | |
| Cohort | Cox | Elley 2013  Mise 2017[90]  Li 2016[28]  Lin 2017[84]  Mise 2016[89]  Fufaa 2015[87]  Foster 2015[86]  Pavkov 2013[92]  Niewczas 2012[91] | 0.883(0.866-0.900)  0.780(0.740-0.820)  0.941(0.887-0.994)  0.910(0.883-0.920)  0.840(0.800-0.880)  0.835(0.828-0.865)  0.845(0.841-0.849)  0.845(0.819-0.871)  0.930(0.890-0.970) | Lin 2017[84]  Wan 2017[29]  Li 2016[28]  Yang 2006[85] | 0.920(0.905-0.934)  0.866(0.849-0.882)  0.882(0.789-0.974)  0.965(0.945-0.985) | Elley 2013[26]  Wan 2017[29]  Basu 2018[44] | 0.918(0.896-0.940)  0.851(0.849-0.853)  0.862(0.860-0.864)  0.780(0.751-0.809)  0.910(0.886-0.934) |
|  |  | Pooled: I^2^ = 92.15 | 0.866(0.842-0.889) | Pooled: I^2^ = 94.86 | 0.912(0.864-0.960) | Pooled: I^2^ = 97.01 | 0.865(0.853-0.877) |
| RCT | Cox | Garlo 2018[27]  Jardine 2012[74]  Heersprink 2010[88] | 0.970(0.946-0.994)  0.847(0.815-0.880)  0.820(0.790-0.860) |  |  |  |  |
|  |  | Pooled: I^2^ = 96.82 | 0.880(0.782-0.978) |  |  |  |  |

### **TABLE S6** Describe variables that were included in the derivative equations

| Study, Year | Prognostic Factors | | | | | | | | | | | | | | | | | | | | | | | | | | | |  |
| --- | --- | --- | --- | --- | --- | --- | --- | --- | --- | --- | --- | --- | --- | --- | --- | --- | --- | --- | --- | --- | --- | --- | --- | --- | --- | --- | --- | --- | --- |
|  | Demographics | | | | Biomarkers | | | | | | | | | | | | | | Clinical Features | | | | | | | | | |  |
|  | Age | Sex | Eth | SDH | Hba1c | TC | HDL | LDL | TG | FPG | UAE | UAC | GFR | SCR | BNP | SUA | Blood | PRO | BMI | SBP | DBP | Smoke | DD | PA | Type | Gen | Waist | Alcohol |  |
| **Diabetic retinopathy (DR)** | | | | | | | | | | | | | | | | | | | | | | | | | | | | | |
|  |  |  |  |  |  |  |  |  |  |  |  |  |  |  |  |  |  |  |  |  |  |  |  |  |  |  |  |  |  |
| Liao 2018[42] | Con | - | - | - | Cat | - | - | - | - | - | - | - | - | - | - | - | - | - | - | Cat | - | - | Cat | - | - | Cat | - | - |  |
| Basu 2017[12] | Con | Cat | Cat | - | Con | Con | Con | - | - | - | - | - | - | Con | - | - | - | - | - | Con | - | - | - | - | - | - | - | - |  |
| Cox 2015[41] | Con | Cat | Cat | Cat | Con | Con | - | - | - | - | - | - | - | - | - | - | - | - | - | Con | - | Cat | Cat | - | Cat | - | - | - |  |
| Oh 2013[17] | Con | - | - | - | Con | - | - | - | Con | Con | - | - | - | - | - | - | Con | - | Con | - | Con | Cat | Con | Cat | - | - | - | Cat |  |
| Wang 2014[47] | Cat | - | - | - | - | - | - | - | - | - | - | - | - | - | - | - | - | - | Cat | - | - | - | Cat | - | - | - | - | - |  |
| Aspelund 2011[11] | Con | Cat | - | - | Con | - | - | - | - | - | - | - | - | - | - | - | - | - | - | Con | - | - | Con | - | Cat | - | - | - |  |
| Hosseini 2009[45] | Cat | Cat | - | - | Cat | - | - | - | - | - | - | - | - | - | - | - | - | - | Cat | - | - | - | Cat | - | - | - | - | - |  |
|  |  |  |  |  |  |  |  |  |  |  |  |  |  |  |  |  |  |  |  |  |  |  |  |  |  |  |  |  |  |
| Mo 2020[49] | Con | - | - | - | Con | - | - | - | - | Con | Con | - | - | Con | - | - | - | - | - | Con | - | - | Con | - | - | - | - | - |  |
| Zhu 2020[53] | - | - | - | - | Con | - | - | Con | - | Con | - | - | - | - | - | - | - | - | - | Con | - | - | Con | - | - | - | - | - |  |
| Ochs 2019[50] | Con | Cat | - | - | Con | Con | - | - | - | - | - | - | - | - | - | - | - | - | Con | Con | Con | Cat | Con | - | - | - | - | - |  |
| Ogunyemi 2019[51] | Con | Cat | Cat | - | Con | - | - | - | - | Cat | - | - | - | - | - | - | - | - | - | - | - | - | Con | - | - | - | - | - |  |
| Tsao 2018[52] | Cat | Cat | - | - | - | - | - | - | - | Cat | - | - | - | - | - | - | - | - | Con | Con | Con |  | Con | Cat | - | - | - | - |  |
| Dagliati 2018[13] | - | - | - | - | Con | - | - | - | - | - | - | - | - | - | - | - | - | - | Con | - | - | Cat | - | - | - | - | - |  |  |
| Finana 2018[48] | - | - | - | - | Con | - | - | - | - | - | - | - | - | - | - | - | - | - | - | Con | - | - | Con | - | Cat | - | - | - |  |
| Herrera 2017[57] | - | - | - | - | - | - | - | - | - | Cat | - | - | - | - | - | - | - | - | - | Cat | - | - | Cat | Cat | - | - | - | - |  |
| Soleiman 2015[54] | Con | Cat | - | Cat | Con | - | - | - | - | Con | - | - | - | - | - | - | - | - | Con | Con | - | - | Con | - | - | - | - | - |  |
| Ogunyemi 2015[15] | Con | Cat | Cat | Cat | Con | - | - | - | - | - | - | - | - | - | - | - | - | - | Cat | - | - | - | Con | - | - | - | - | Cat |  |
| Ogunyemi 2013[16] | Con | Cat | Cat | Cat | Con | - | - | - | - | - | - | - | - | - | - | - | - | - | Cat | - | - | - | Con | - | - | - | - |  |  |
| Tanaka 2013[59] | Con | - | - | - | Con | - | - | - | - | - | - | Con | - | - | - | - | - | - | Cat | - | - | - | Con | - | - | - | - | - |  |
| Semeraro 2011[18] | Con | Cat | - | - | Con | - | - | - | - | - | - | - | - | Con | - | - | - | - | - | Con | - | - | Con | - | - | - | - | - |  |
| Ng 2008[58] | - | - | - | - | - | - | - | - | - | Con | - | - | - | - | - | - | - | - | - | - | - | - | Con | - | Cat | - | - | - |  |
| Han 2004[56] | - | - | - | - | - | - | - | - | - | Con | - | - | - | - | - | - | - | - | - | - | - | - | Con | - | Cat | - | - | - |  |
|  |  |  |  |  |  |  |  |  |  |  |  |  |  |  |  |  |  |  |  |  |  |  |  |  |  |  |  |  |  |
| Chong 2017[55] | Con | Cat | Cat | - | Con | - | - | - | - | - | - | - | - | - | - | - | - | - | - | - | - | - | Con | - | - | Cat | - | - |  |
| Mogi 2017[62] | - | - | - | - | - | - | - | - | - | - | - | - | - | - | - | - | - | - | - | - | - | - | - | - | - | Cat | - | - |  |
| Kengne 2015[14] | Con | - | - | - | - | - | - | - | - | Con | - | - | - | - | - | - | - | - | - | - | - | - | Con | - | - | - | - | - |  |
| Cichosz 2015[61] | Con |  | - | - | Con | - | - | - | - | - | Con | Con | - | - | - | - | - | - | Con | Con | - | - | - | - | - | - | Con | - |  |
| Welsh 2014[24] | - | - | - | Cat | Con | Con | Con | - | Con | Con | - | Con | Con | - | Con | - | - | Con | Con | Con | - | Cat | Con | - | - | - | - | - |  |
| Bresnick 1987[60] | Con | - | - | - | - | - | - | - | - | - | - | - | - | - | - | - | - | - | - | - | - | - | Con | - | - | - | - | - |  |
| Subtotal | 20 | 12 | 6 | 5 | 20 | 4 | 2 | 1 | 2 | 11 | 2 | 3 | 1 | 3 | 1 | 0 | 1 | 1 | 12 | 14 | 3 | 5 | 24 | 3 | 5 | 3 | 1 | 2 |  |
| **Chronic Kidney Diseases (CKD)** | | | | | | | | | | | | | | | | | | | | | | | | | | | | | |
|  |  |  |  |  |  |  |  |  |  |  |  |  |  |  |  |  |  |  |  |  |  |  |  |  |  |  |  |  |  |
| Song 2019[71] | Con | Cat | Cat | Cat | Con | Con | Con | Con | Con | Con | Con | Con | Con | Con | Con | Con | Con | Con | Con | Con | Con | Cat | Con | Con | Con | - | Con | - |  |
| Peters 2019[68] | Con | - | - | - | - | - | Con | - | - | - | - | - | Con | - | - | - | - | Con | - | - | - | - | - | - | - | - | - | - |  |
| Nowak 2018[23] | - | - | - | - | - | - | - | - | - | - | - | Con | - | - | - | - | - | Con | - | Con | - | - | - | - | - | - | - | - |  |
| Basu 2017[12] | Con | Cat | Cat | - | Con | Con | Con | - | - | - | - | - | - | Con | - | - | - | - | - | Con | - | Cat | - | - | - | - | - | - |  |
| Blech 2011[19] | Con | Cat | Cat | - | - | - | - | - | - | - | - | - | - | - | - | - | - | - | - | - | - | - | Con | - | Cat | Cat | - | - |  |
| Miao 2017[22] | Con | - | - | - | - | - | Con | - | - | - | - | - | - | Con | - | - | - | - | Con | - | - | - |  | Cat | - | - | - | - |  |
| Wu 2017[25] | - | Cat | - | - | - | - | - | - | - | - | - | - | - | - | - | - | - | - | Cat | Cat | - | - | Cat | - | - | - | - | - |  |
| Dunkler 2015[20] | Con | - | - | - | - | - | - | - | - | - | - | Con | Con | - | - | - | - | - | - | - | - | - | - | - | - | - | - | - |  |
|  |  |  |  |  |  |  |  |  |  |  |  |  |  |  |  |  |  |  |  |  |  |  |  |  |  |  |  |  |  |
| Hu 2020 | Cat | Cat | - | - | Cat | - | - | - | Cat | Cat | - | - | - | Cat | - | - | - | - | Cat | Cat | Cat | Cat | Cat | - | - | - | - | - |  |
| Wysham 2020[72] | Cat | Cat | Cat | Cat | - | - | - | - | - | - | - | - | - | - | - | - | - | - | - | - | - | - | Cat | - | - | - | - | - |  |
| Jiang 2020[66] | - | Cat | - | - | Cat | - | - | - | - | - | Cat | - | Cat | - | - | - | Cat | - | - | Cat | Cat | - | Cat | - | - | - | - | - |  |
| Liao 2019[67] | Con | Cat | - | - | - | - | - | - | Cat | - | - | - | - | - | - | - | - | - | Cat | - | - | - | - | - | - | Cat | - | - |  |
| Romero 2019[70] | Con | - | - | - | - | Con | Con | Con | Con | Con |  | Con | Con | Con | - | - | - | Con | - | Con | Con | - | - | - | - | - | - | - |  |
| Dagliati 2018[13] | - | - | - | - | Con | - | - | - | - | - | - | - | - | - | - | - | - | - | Con | - | - | Cat | - | - | - | - | - | - |  |
| Low 2017[78] | Con | - | - | - | Con | - | - | Con | - | - | - | Con | Con | - | - | - | - | - | - | Con | - | - | - | - | - | - | - | - |  |
| Peters 2017[79] | Con | - | - | - | - | - | Con | - | - | - | - | - | - | - | - | - | - | Con | - | - | - | - | Con | - | - | - | - | - |  |
| Tanaka 2013[59] | - | - | - | - | Con | - | - | - | - | - | - | Con | - | - | - | - | - | - | - | Con | - | Cat | - | - | - | - | - | - |  |
| Jardine 2012[74] | - | - | Cat | - | Con | - | - | - | - | - | - | Con | Con | - | - | - | - | - | - | Con | - | - | - | - | - | - | Con | - |  |
| Goldfarb 2002[73] | Con | - | - | - | - | - | - | Con | - | - | Con | Con | Con | Con | - | - | - | Con | - | Con | Con | - | Con | - | - | - | - | - |  |
|  |  |  |  |  |  |  |  |  |  |  |  |  |  |  |  |  |  |  |  |  |  |  |  |  |  |  |  |  |  |
| Klisic 2018[21] | - | - | - | - | - | - | - | - | Con |  | Con | - | - | - | - | - | - | Con | Con | - | - | - | - | - | - | - | - | - |  |
| Jenks 2017[75] | Con | Cat | - | - | Con | - | - | - | - | - | - | Con | Con | - | - | - | - | Con | - | - | - | Cat | Con | - | - | - | - | - |  |
| Kim 2017[76] | Con | Cat | - | - | Con | - | - | Con | - | - | - | Con | Con | - | - | - | - | - | - | Con | - | - | Con | - | - | - | - | - |  |
| Lindhart 2017[77] | Con | Cat | - | - | Con | - | Con | - | - | - | Con | - | Con | - | - | - | - | Con | - | Con | - | Cat |  | - | - | - | - | - |  |
| Saulnier 2017[81] | Con | Cat | - | - | Con | - | - | - | - | - | - | Con | Con | - | Con | - | - | Con | - | Con | - | - | Con | - | - | - | - | - |  |
| Yang 2017[82] | Con | Cat | - | - | Con | - | - | - | - | - | Con | - | Con | - | - | - | - | - | Con | Con | - | - | Con | - | - | - | - | - |  |
| Zobel 2017[83] | Con | Cat | - | - | Con | - | - | Con | - | - | Con | - | - | Con | - | - | - | - | - | - | - | Cat | - | - | - | - | - | - |  |
| Riphagen 2015[80] | Con | Cat | - | - | Con | - | - | - | - | - | - | Con | - | - | - | - | - | - | Con | Con | - | Cat | - | - | - | - | - | - |  |
| Welsh 2014[24] | - | - | - | Cat | Con | Con | Con |  | Con | Con | - | Con | Con | - | Con |  | Con | - | Con | Con | - | Cat | Con | - | - | - | - | - |  |
| Subtotal | 20 | 15 | 5 | 3 | 16 | 4 | 8 | 6 | 6 | 4 | 7 | 13 | 14 | 7 | 3 | 1 | 3 | 10 | 10 | 17 | 5 | 10 | 13 | 2 | 2 | 2 | 2 | 0 |  |
| **End stage renal disease (ESRD)** | | | | | | | | | | | | | | | | | | | | | | | | | | | | | |
|  |  |  |  |  |  |  |  |  |  |  |  |  |  |  |  |  |  |  |  |  |  |  |  |  |  |  |  |  |  |
| Basu 2017[12] | Con | Cat | Cat | - | Con | Con | Con | - | - | - | - | Con | - | Con | - | - | - | - | - | Con | - | Cat | - | - | - | - | - | - |  |
| Wan 2017[29] | Con | - | - | - | Con | - | - | - | - | - | - | Con | Con | - | - | - | - | - | - | Con | Con | Cat | - | - | - | - | - | - |  |
| Elley 2013[26] | Con | Cat | Cat | - | Con | Con | - | - | - | - | - | - | - | Con | - | - | - | - | Con | Con | - | Cat | Con | - | - | - | - | - |  |
|  |  |  |  |  |  |  |  |  |  |  |  |  |  |  |  |  |  |  |  |  |  |  |  |  |  |  |  |  |  |
| Wysham 2020[72] | Cat | Cat | Cat | Cat | - | - | - | - | - | - | - | - | - | - | - | - | - | - | - | - | - | - | Cat | - | - | - | - | - |  |
| Lin 2017[84] | Cat | Cat | - | - | Cat | - | - | - | - | - | - | - | - | Cat | - | - | - | - | - | Cat | - | - | Cat | - | - | - | - | - |  |
| Li 2016[28] | - | - | - | - | Con | - | - | - | - | - | - | Con | Con | - | - | - | - | Con | - | - | - | - | - | - | - | - | - | - |  |
| Jardine 2012[74] | Cat | Cat | - | - | Con | - | - | - | - | - | - | Con | Con | - | - | - | - | - | - | Con | - | - | - | - | - | - | - | - |  |
| Yang 2006[85] | - | - | - | - | - | Con | Con | - | - | - | - | - | Con | - | - | - | - | - | - | Con | - | - | Con | - | - | - | - | - |  |
|  |  |  |  |  |  |  |  |  |  |  |  |  |  |  |  |  |  |  |  |  |  |  |  |  |  |  |  |  |  |
| Garlo 2018[27] | Con | Cat | Cat | - | Con | - | - | - | - | - | - | Con | Con | - | - | - | - | Con | - | Con | - | - | - | - | - | - | - | - |  |
| Mise 2017[90] | Con | Cat | - | - | - | - | - | - | - | - | Con | - | Con | - | - | - | - | - | Con | Con | - | - | Con | - | - | - | - | - |  |
| Mise 2016[89] | Con | Cat | - | - | - | - | - | - | - | - | Con | - | Con | - | - | - | - | - | Con | Con | - | - | - | - | - | - | - | - |  |
| Foster 2015[86] | Con | Cat | - | - | Con | Con | - | - | - | - | - | Con | Con | - | - | - | - | Con | - | - | - | - | Con | - | - | - | - | - |  |
| Fufaa 2015[87] | Con | Cat | - | - | Con | - | - | - | - | - | - | Con | Con | - | - | - | - | Con | - | - | - | - | Con | - | - | - | - | - |  |
| Pavkov 2013[92] | Con | Cat | - | - | Con | - | - | - | - | - | - | - | - | Con | - | - | - | - | Con | - | - | - | Con | - | - | - | - | - |  |
| Niewczas 2012[91] | - | - | - | - | Con | - | - | - | - | - | Con | - | Con | - | - | - | - | Con | - | - | - | - | - | - | - | - | - | - |  |
| Heersprink 2010[88] | Con | Cat | Cat | - | - | - | - | - | - | - | - | - | - | - | - | - | - | Con | - | - | - | - | - | - | - | - | - | - |  |
| Keane 2006[93] | - | - | - | - | Con | - | - | - | - | - | Con | Con | - | Con |  |  | Con | - | - | - | - | - | - | - | - | - | - | - |  |
| Subtotal | 13 | 12 | 5 | 1 | 12 | 4 | 2 | 0 | 0 | 0 | 4 | 8 | 10 | 5 | 0 | 0 | 1 | 6 | 4 | 9 | 1 | 3 | 8 | 0 | 0 | 0 | 0 | 0 |  |
| Total | 53 | 39 | 16 | 9 | 49 | 12 | 12 | 7 | 8 | 15 | 13 | 24 | 25 | 16 | 4 | 1 | 6 | 17 | 26 | 41 | 9 | 18 | 46 | 5 | 7 | 5 | 3 | 2 |  |

### **TABLE S6** Continued

| Study, Year | Prognostic factors | | | | | | | | | | | | | | |
| --- | --- | --- | --- | --- | --- | --- | --- | --- | --- | --- | --- | --- | --- | --- | --- |
|  |  | Drug Usage | | | | | Comorbidities | | | | | | | | Image/Signal Process |
|  | FHD | BP-drug | Oral diabetic | Insulin | Statins | Anti-coagulant | HT | CVD | DLP | PN | DR | PAD | DN | Other disease |  |
| **Diabetic Retinopathy** | | | | | | | | | | | | | | | |
|  |  |  |  |  |  |  |  |  |  |  |  |  |  |  |  |
| Liao 2018[42] | - | - | - | - | - | - | - | - | - | - | - | - | - | - | - |
| Basu 2017[12] | - | Cat | Cat | - | - | - | - | Cat | - | - | - | - | - | - | - |
| Cox 2015[41] | - | - | - | - | - | - | - | - | - | - | Cat | - | Cat | - | - |
| Oh 2013[17] | - | - | - | Cat | Cat | - | - | - | - | - | - | - | - | - | - |
| Wang 2014[47] | - | Cat | - | - | - | - | - | - | - | - | - | - | - | - | - |
| Aspelund 2011[11] | - | - | - | - | - | - | - | - | - | - | - | - | - | - | - |
| Hosseini 2009[45] | - | - | - | - | - | - | - | - | - | - | - | - | - | - | - |
|  |  |  |  |  |  |  |  |  |  |  |  |  |  |  |  |
| Mo 2020[49] | - | - | - | - | - | - | - | - | - | - | - | - | - | - | - |
| Zhu 2020[53] | - | - | - | - | - | - | - | - | - | - | - | - | - | - | - |
| Ochs 2019[50] | - | - | - | - | - | - | - | - | - | - | - | - | - | - | - |
| Ogunyemi 2019[51] | - | - | - | Cat | - | - | - | - | - | - | - | - | - | - | - |
| Tsao 2018[52] | Cat | - | - | Cat | - | - | - | - | - | - | - | - | - | - | - |
| Dagliati 2018[13] | - | - | - | - | - | - | Cat | - | - | - | - | - | - | - | - |
| Finana 2018[48] | - | - | - | - | - | - | - | - | - | - | - | - | - | - | - |
| Herrera 2017[57] | - | - | - | - | - | - | - | - | - | - | - | - | - | - | - |
| Soleiman 2015[54] | - | - | - | - | - | - | - | - | - | - | - | - | Cat | - | - |
| Ogunyemi 2015[15] | - | - | - | Cat | - | - | Cat | Cat | Cat | Cat | Cat | Cat | Cat | Cat | - |
| Ogunyemi 2013[16] | - | - | Cat | - | - | - | Cat | Cat | Cat | Cat | Cat | Cat | Cat | Cat | - |
| Tanaka 2013[59] | - | - | Cat | - | - | - | - | - | - | - | - | - | - | - | - |
| Semeraro 2011[18] | - | - | Cat | - | - | - | - | - | - | - | - | - | - | - | - |
| Ng 2008[58] | - | - | - | - | - | - | - | - | - | - | - | - | - | - | Con |
| Han 2004[56] | - | - | - | - | - | - | - | - | - | - | Cat | - | - | - | Con |
|  |  |  |  |  |  |  |  |  |  |  |  |  |  |  |  |
| Chong 2017[55] | - | - | - | - | - | - | Cat | - | - | - | - | - | - | - | - |
| Mogi 2017[62] | - | - | - | - | - | - | - | - | - | - | - | - | - | - | - |
| Kengne 2015[14] | - | - | Cat | - | - | - | Cat | - | - | Cat | - | - | Cat | - | - |
| Cichosz 2015[61] | - | - | - | - | - | - | - | - | - | - | - | - | - | - | - |
| Welsh 2014[24] | - | - | - | - | - | - | - | Cat | - | - | - | - | - | - | - |
| Bresnick 1987[60] | - | - | - | - | - | - | - | - | - | - | Cat | - | - | - | - |
| Subtotal | 1 | 2 | 5 | 4 | 1 | 0 | 5 | 4 | 2 | 3 | 5 | 2 | 5 | 2 | 2 |
| **Chronic Kidney Disease (CKD)** | | | | | | | | | | | | | | | |
|  |  |  |  |  |  |  |  |  |  |  |  |  |  |  |  |
| Song 2019[71] | - | Cat | Cat | Cat | - | - | Cat | Cat | Cat | - | - | - | - | Cat | - |
| Peters 2019[68] | - | - | - | - | - | - | - | - | - | - | - | - | - | - | - |
| Nowak 2018[23] | - | - | - | - | - | - | - | - | - | - | - | - | Cat | - | - |
| Basu 2017[12] | - | Cat | Cat | - | - | Cat | - | - | - | - | - | - | - | - | - |
| Blech 2011[19] | - | - | - | - | - | - | - | - | - | - | - | - | - | - | - |
| Miao 2017[22] | - | - | - | - | - | - | Cat | - | - | - | Cat | - | - | - | - |
| Wu 2017[25] | - | - | - | - | - | - | - | - | - | - | - | - | - | - | - |
| Dunkler 2015[20] | - | - | - | - | - | - | - | - | - | - | - | - | - | - | - |
|  |  |  |  |  |  |  |  |  |  |  |  |  |  |  |  |
| Hu 2020[65] | - | - | - | - | - | - | - | - | - | - | - | - | - | - | - |
| Wysham 2020[72] | - | - | - | - | - | - | Cat | Cat | - | - | - | - | Cat | Cat | - |
| Jiang 2020[66] | - | - | - | - | - | - | - | - | - | - | Cat | - | - | - | - |
| Liao 2019[67] | - | - | - | - | - | - | Cat | Cat | - | - | - | - | - | - | - |
| Romero 2019[70] | - | - | - | - | - | - | - | Cat | - | - | - | - | - | - | - |
| Dagliati 2018[13] | - | - | - | - | - | - | Cat | - | - | - | - | - | - | - | - |
| Low 2017[78] | - | - | - | - | - | - | - | - | - | - | - | - | - | - | - |
| Peters 2017[79] | - | Cat | - | - | - | - | - | - | - | - | - | - | - | - | - |
| Tanaka 2013[59] | - | - | - | - | - | - | - | Cat | - | - | - | - | - | - | - |
| Jardine 2012[74] | - | Cat | - | - | - | - | - | - | - | - | Cat | - | - | - | - |
| Goldfarb 2002[73] | - | - | - | - | - | - | - | - | - | - | - | - | - | - | - |
|  |  |  |  |  |  |  |  |  |  |  |  |  |  |  |  |
| Klisic 2018[21] | - | - | - | - | - | - | - | - | - | - | - | - | - | - | - |
| Jenks 2017[75] | - | - | - | - | - | - | - | Cat | - | - | Cat | - | - | - | - |
| Kim 2017[76] | - | Cat | Cat | - | - | - | - | - | - | - | - | - | - | - | - |
| Lindhart 2017[77] | - | Cat | - | - | - | - | - | - | - | - | - | - | - | - | - |
| Saulnier 2017[81] | - | - | - | - | - | - | - | - | - | - | - | - | - | - | - |
| Yang 2017[82] | - | - | - | - | - | - | - | - | - | - | - | - | Cat | - | - |
| Zobel 2017[83] | - | - | - | - | - | - | - | - | - | - | - | - | - | - | - |
| Riphagen 2015[80] | - | - | - | - | - | - | - | Cat | - | - | - | - | - | - | - |
| Welsh 2014[24] | - | - | - | - | - | - | - | Cat | - | - | - | - | - | - | - |
| Subtotal | 0 | 6 | 3 | 1 | 0 | 1 | 5 | 8 | 1 | 0 | 4 | 0 | 3 | 2 | 0 |
| **End Stage Renal Disease (ESRD)** | | | | | | | | | | | | | | | |
|  |  |  |  |  |  |  |  |  |  |  |  |  |  |  |  |
| Basu 2017[12] | - | Cat | Cat | - | - | Cat | - | Cat | - | - | - | - | - | - | - |
| Wan 2017[29] | - | - | Cat | Cat | - | Cat | - | - | - | - | Cat | - | - | - | - |
| Elley 2013[26] | - | - | - | - | - | - | - | Cat | - | - | - | - | Cat | - | - |
|  |  |  |  |  |  |  |  |  |  |  |  |  |  |  |  |
| Wysham 2020[72] | - | - | - | - | - | - | Cat | Cat | - | - | - | - | Cat | Cat | - |
| Lin 2017[84] | - | Cat | Cat | - | - | - | - | - | - | - | Cat | - | Cat | - | - |
| Li 2016[28] | - | - | - | - | - | - | - | - | - | - | - | - | Cat | - | - |
| Jardine 2012[74] | - | - | - | - | - | - | - | - | - | - | Cat | - | - | - | - |
| Yang 2006[85] | - | Cat | - | - | - | - | - | - | - | - | Cat | - | - | - | - |
|  |  |  |  |  |  |  |  |  |  |  |  |  |  |  |  |
| Garlo 2018[27] | - | - | - | - | - | - | Cat | - | - | - | - | - | Cat | - | - |
| Mise 2017[90] | - | - | - | - | - | - | - | - | - | - | Cat | - | Cat | - | - |
| Mise 2016[89] | - | - | - | - | - | - | - | - | - | - | Cat | - | - | - | - |
| Foster 2015[86] | - | - | - | - | - | - | Cat | - | - | - | - | - | - | - | - |
| Fufaa 2015[87] | - | - | - | - | - | - | Cat | - | - | - | - | - | - | - | - |
| Pavkov 2013[92] | - | - | - | - | - | - | - | - | - | - | - | - | - | - | - |
| Niewczas 2012[91] | - | - | - | - | - | - | - | - | - | - | - | - | - | - | - |
| Heersprink 2010[88] | - | - | - | - | - | - | - | - | - | - | - | - | - | - | - |
| Keane 2006[93] | - | - | - | - | - | - | - | - | - | - | - | - | - | - | - |
| Subtotal | 0 | 3 | 3 | 1 | 0 | 2 | 4 | 4 | 0 | 0 | 6 | 0 | 6 | 1 | 0 |
| **Total** | 1 | 11 | 11 | 6 | 1 | 3 | 14 | 16 | 3 | 3 | 15 | 2 | 14 | 5 | 2 |

**Abbreviations:** ALC, Alcohol consumption; BMI, Body mass index; BNP, Brain Natriuretic Peptide; BP-drug, Blood pressure-drug; Cat, categorical forms; Con, Continuous forms; CVDs, cardiovascular diseases; DBP, Diastolic blood pressure; DD, diabetic duration; DLP, Dyslipidemia; DN, diabetic nephropathy; DR, Diabetic retinopathy; e-GFR, Estimated Glomerular Filtration Rate; Eth, Ethnicity; FHD, family history of diabetes; FPG, Fasting plasma glucose; Gen, Genetic factors; HDL, High-density lipoprotein; HT, Hypertension; LDL, Low density lipoprotein; PA, Physical activity; PN, Peripheral neuropathy; PRO, protein; PAD, peripheral arterial disease; SBP, Systolic blood pressure; S-Cr, Serum creatinine; SDH, Social determinant of health (i.e., education, income, region, etc.); SUA, Serum uric acid; TC, Total cholesterol; TC, Total cholesterol; TG, Triglycerides; UAC, Urine albumin creatinine; UAE, Urine albumin excretion; WC, Waist circumference
